# TRIM25 promotes antiviral innate immune response by stabilizing IRF7 and its nuclear translocation

**DOI:** 10.1128/mbio.00470-26

**Published:** 2026-04-01

**Authors:** Zhen Wu, Yingxuan Cui, Yu He, Tao Wang, Tao Hu, Mingshu Wang, Renyong Jia, Dekang Zhu, Mafeng Liu, Xinxin Zhao, Qiao Yang, Ying Wu, Shaqiu Zhang, Juan Huang, Xumin Ou, Di Sun, Bin Tian, Anchun Cheng, Shun Chen

**Affiliations:** 1 Institute of Veterinary Medicine and Immunology, Sichuan Agricultural University, Chengdu, Sichuan, China; 2 Research Center of Avian Disease, College of Veterinary Medicine, Sichuan Agricultural University 506176 , Chengdu, Sichuan, China; 3 Agricultural Animal Diseases and Veterinary Public Health Key Laboratory of Sichuan Province, Sichuan Agricultural University 506176 , Chengdu, Sichuan, China; 4 Key Laboratory of Agricultural Bioinformatics, Ministry of Education, Sichuan Agricultural University 506176 , Chengdu, Sichuan, China; 5 Engineering Research Center of Southwest Animal Disease Prevention and Control Technology for Ministry of Education, Sichuan Agricultural University 506176 , Chengdu, Sichuan, China; 6 Veterinary Department in College of Animal Science, State Key Laboratory of Green Pesticide, Institute of Veterinary Immunology and Green Drugs, Guizhou University 71206, Guiyang, China; Huazhong Agricultural University, Wuhan, Hubei, China

**Keywords:** TRIM25, IRF7, innate immunity

## Abstract

Most avian species lack interferon regulatory factor 3 (IRF3), a key transcription factor that initiates antiviral interferon responses, leaving how they achieve rapid RNA virus defense unclear. Here, we identify duck tripartite motif containing 25 protein (TRIM25) as an unexpected, central activator of IRF7-dependent antiviral signaling that compensates for the absence of IRF3. Viral RNA stimulation strongly induced TRIM25 expression in duck embryonic fibroblasts (DEFs). Overexpression of TRIM25 increased IFN-β production and restricted the replication of multiple RNA viruses in DEFs and in ducklings, whereas TRIM25 knockdown enhanced viral replication (tembusu virus, TMUV, novel duck reovirus, NDRV, vesicular stomatitis virus, VSV). Mechanistically, TRIM25 functioned as an E3 ubiquitin ligase that catalyzed K27-linked polyubiquitination of IRF7 at lysine 46 (K46), thereby promoting IRF7 stability and nuclear translocation. Mutation of TRIM25 catalytic residues or of IRF7 K46 abolished these effects. In addition, TRIM25 stabilized IRF7 independently of its ligase activity by antagonizing suppressor of cytokine signaling 1 (SOCS1)-mediated degradation. Notably, TRIM25-driven signaling occurred independently of retinoic acid-inducible gene I (RIG-I) activation. Together, these findings reveal a dual regulatory mechanism by which avian TRIM25 activates IRF7 to elicit robust type I interferon (IFN-I) responses, providing a molecular explanation for rapid antiviral immunity in species lacking IRF3.

**IMPORTANCE:**

Most avian species, including those in the orders *Galliformes* and *Anseriformes*, lack IRF3; consequently, IRF7 plays a pivotal and more comprehensive role in regulating innate immunity against viral infections. However, the activation and regulation by IRF7 in avian remain insufficiently characterized. In this study, we identify the E3 ubiquitin ligase duck TRIM25 as a positive regulator of IRF7. Functioning as an E3 ligase, TRIM25 stabilizes IRF7 expression and promotes its nuclear translocation through K27-linked polyubiquitination, thereby enhancing IFN-I production. Additionally, TRIM25 stabilizes IRF7 independently of its E3 ligase activity by suppressing SOCS1-mediated degradation. Activation of TRIM25 *in vivo* in ducks exhibits significant efficacy in antagonizing RNA virus infection. Collectively, our findings demonstrate that TRIM25 functions as a positive regulator, promoting IRF7-dependent signaling pathways to combat RNA viruses. This establishes TRIM25 as a crucial regulatory factor and potential target within the antiviral response.

## INTRODUCTION

The activation of the IFN-I signaling cascade follows a strictly regulated sequence. Following viral infection, pattern recognition receptors such as RIG-I and melanoma differentiation-associated gene 5 (MDA5) detect viral RNA within the cell. A critical step preceding IFN production is the phosphorylation of transcription factors—including IRF3, IRF7, and nuclear factor kappa-B (NF-κB)—and their subsequent translocation to the nucleus (1–4). The innate immune architecture of avian species exhibits significant specialized adaptations that diverge from mammalian paradigms. While the IRF3 molecule is broadly conserved across diverse vertebrate lineages—extending from mammals to more anciently diverged groups such as teleost fish—comparative genomic analyses reveal a more nuanced evolutionary history within the avian class. Rather than a universal absence, it appears that IRF3 has undergone a lineage-specific secondary loss in the majority of avian species. Notably, recent evidence indicates that certain basal avian lineages, such as the emu (*Dromaius novaehollandiae*), have retained a functional IRF3 ortholog (5). Current evidence suggests that IRF7 may functionally compensate for the absence of IRF3. In avian, IRF7 is transcriptionally upregulated upon viral infection and phosphorylated by TANK binding kinase 1 (TBK1), mirroring the role of IRF3 in mammals, though the precise mechanisms remain incompletely understood (6, 7). Avian IRF7 has been shown to mediate innate immune responses against both RNA and DNA viruses. Studies in chickens further reveal that the adaptor protein stimulator of interferon genes (STING) serves as a scaffold that bridges IRF7 and TBK1, facilitating IRF7 phosphorylation, homodimerization, nuclear translocation, and ultimately driving IFN-I synthesis (6).

In mammals, IRF3 acts as a “first responder,” initiating rapid interferon expression during the early phase of infection, whereas IRF7 serves as the “reinforcement and amplification unit,” whose expression is tightly regulated to amplify and sustain the interferon response (8, 9). IRF3 is constitutively and widely expressed across many cell types, forming the basis for its role as a key rapid-response molecule in innate immunity (8). In contrast, IRF7 expression is highly inducible, predominantly restricted to lymphoid tissues, and present at minimal levels under basal conditions (10). The constitutive expression and rapid-response function of avian IRF7 in cells constitute a fundamental basis for its functional substitution for IRF3 in avian (11, 12). This adaptation highlights an evolutionary strategy, wherein IRF7 has assumed the early-response role typically associated with IRF3 in mammals, enabling an effective initial antiviral defense in the absence of IRF3.

IRF7 undergoes diverse post-translational modifications—including phosphorylation (13), ubiquitination (14), acetylation (15), SUMOylation (16), and NEDDylation (17)—that dynamically regulate its stability and activity (18). Among these, ubiquitination plays a pivotal role in maintaining immune equilibrium by modulating IRF7 turnover and signaling. The TRIM protein family, comprising at least 80 members in most eukaryotes, including birds, is defined by three conserved domains: an N-terminal really interesting new gene (RING) finger domain, one or two B-box domains, and a coiled-coil (CC) region, collectively designated the RBCC motif (19). The RING domain confers E3 ubiquitin ligase activity, enabling TRIM proteins to execute substrate ubiquitination critical for innate immune regulation and antiviral responses (20–22). For instance, FAS-associated death domain protein (FADD) recruits TRIM21, thereby promoting the ubiquitination and degradation of IRF7 and negatively regulating the IFN-I pathway (14). Human TRIM14 is localized in mitochondria and can promote the activation of IRF3 and NF-κB mediated by RIG-I-like receptors, serving as a mediator of mitochondrial antiviral immunity (23). Duck TRIM14 combats TMUV by catalyzing K27/K29-linked polyubiquitination of viral nonstructural protein 1 (NS1), targeting it for proteasomal degradation, while simultaneously enhancing RLR signaling via K63-linked polyubiquitination of duck TBK1 (24). Thus, TRIM proteins precisely regulate the stability and function of key signaling molecules like IRF7 through various ubiquitination modifications, representing important immune regulators.

Among the numerous TRIM family members, TRIM25’s function is relatively well-defined in mammalian systems, but its role in avian, particularly regarding IRF7 regulation, remains largely unexplored. In mammalian systems, TRIM25 is extensively studied and well-established as a crucial E3 ubiquitin ligase. It catalyzes K63-linked ubiquitination of RIG-I, facilitating its interaction with MAVS and thereby amplifying antiviral signaling (25). However, significant differences between avian and mammalian immune systems suggest that avian TRIM proteins may have evolved distinct functions. This study, therefore, investigates whether, and by what mechanisms, duck TRIM25 regulates IRF7, aiming to reveal its unique contribution to the avian antiviral defense.

In this study, we uncover that duck TRIM25 stabilizes IRF7 expression and facilitates its nuclear translocation through two novel mechanistically distinct pathways. E3 ligase-dependent stabilization: TRIM25 employs its intrinsic E3 ubiquitin ligase activity to catalyze K27-linked polyubiquitination of IRF7, thereby enhancing its stability. E3 ligase-independent stabilization: beyond its catalytic function, TRIM25 also prevents SOCS1-mediated degradation of IRF7, even in its catalytically inactive mutant form. Collectively, both mechanisms potentiate IFN-I production, effectively restricting RNA viral replication.

## RESULTS

### Duck TRIM25 modulates duck innate immunity via a RIG-I-independent pathway

To investigate the differences between duck TRIM25 and its mammalian counterpart in promoting IFN-I production and its interaction with RIG-I, we examined their interplay in both chicken and duck cells. The results showed that duck TRIM25 induces RIG-I ubiquitination and IFN production in DF-1 cells (chicken) (Fig. S1A and B), which was consistent with previous studies (26). Critically, overexpression of TRIM25 in DEFs did not enhance duck RIG-I-mediated IFN-I mRNA induction (Fig. S1C). Consistently, TRIM25 failed to promote the ubiquitination of duck RIG-I in DEFs (Fig. S1D), and similar results were observed in the dual-luciferase reporter assays, where no significant enhancement of RIG-I-mediated signaling was detected (Fig. S1E). These data collectively demonstrate that TRIM25 does not regulate RIG-I-induced IFN production in DEFs.

### TRIM25 promotes IFN production in response to RNA viral infection

**Fig 1:**
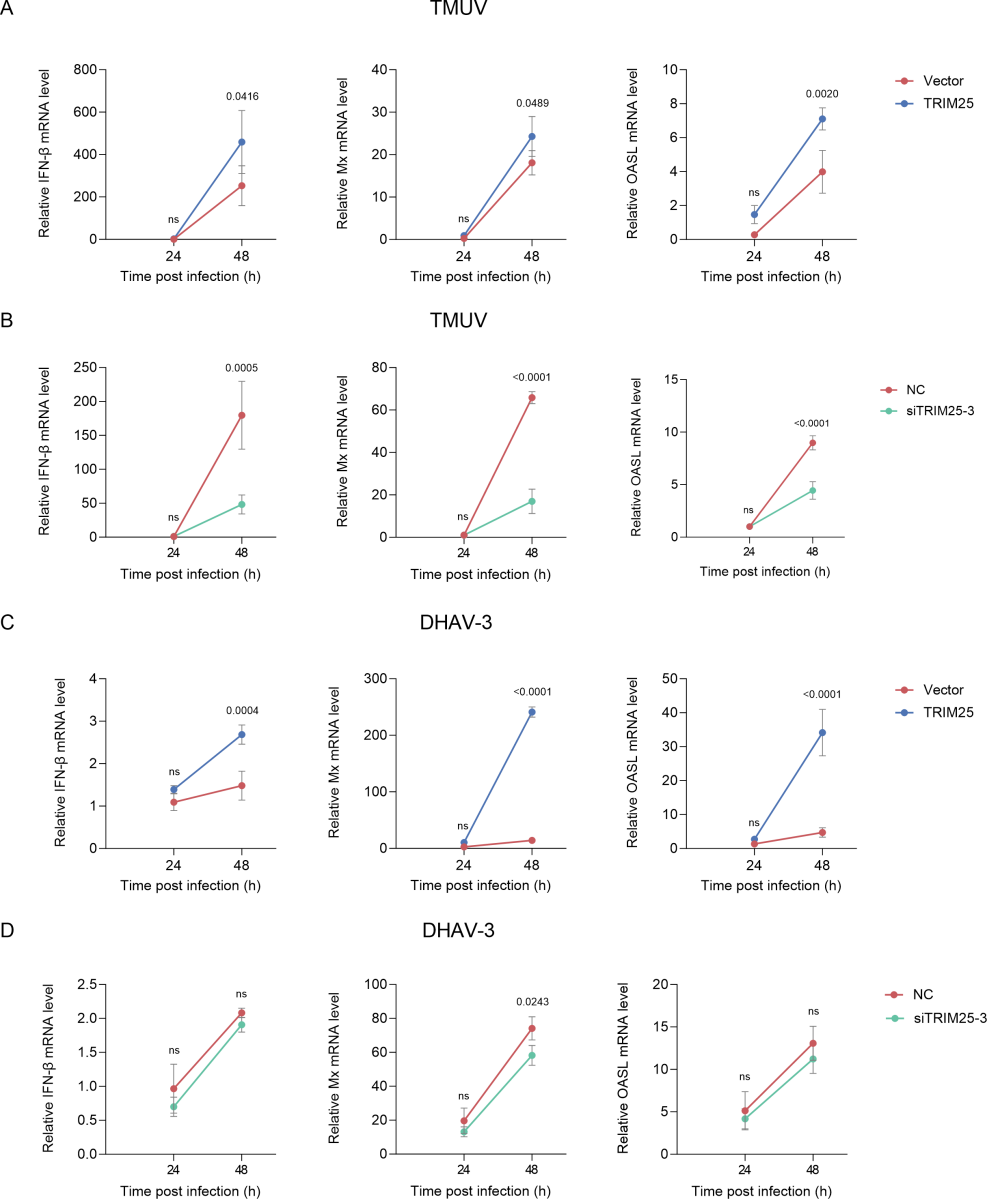
TRIM25 inhibits type I IFN production during RNA virus infection. (**A–D**) DEFs were transfected with TRIM25 (**A and C**) or siTRIM25 (**B and D**) for 24 h, followed by TMUV (2,000 TCID_50_; **A and B**) or DHAV-3 (30 μL; **C and D**) infection. IFNβ, Mx, and OASL mRNA levels were determined by RT-qPCR (mean ± SD, *n* = 3). Statistical analysis was performed using one-way ANOVA with Dunnett’s multiple comparisons test.

To test if TRIM25 modulates RNA virus replication via the IFN-I pathway, we transfected DEFs with TRIM25 expression plasmid or siRNA, challenged them with various RNA viruses, including TMUV and duck hepatitis A virus genotype 3 (DHAV-3), and measured the transcription of IFN-β and its downstream interferon stimulation genes (ISGs) (Mx and OASL). Western blot analysis confirmed efficient TRIM25 knockdown in DEFs transfected with three distinct siRNAs, among which siTRIM25-3 exhibited the strongest silencing efficiency and was, therefore, used in subsequent experiments (Fig. S2). As expected, TRIM25 overexpression significantly enhanced, whereas TRIM25 knockdown substantially reduced the mRNA levels of IFN-β, Mx, and OASL in TMUV-infected DEFs at 48 h post-infection (hpi), compared to vector controls (Fig. 1A and B). Similarly, in DHAV-3-infected cells, TRIM25 overexpression significantly upregulated the transcription of IFN-β, Mx, and OASL compared to vector controls (Fig. 1C). Conversely, TRIM25 knockdown notably reduced Mx expression, while IFN-β and OASL transcripts exhibited consistent downward trends, although the decreases were not statistically significant (Fig. 1D). Given the potent induction of avian innate immunity by RNA viruses, NDRV and VSV, we reconfirmed TRIM25’s regulatory role in DEFs. TRIM25 overexpression significantly enhanced NDRV- or VSV-induced expression of IFN-β, Mx, and OASL relative to vector controls (Fig. S3A and B). These results demonstrate that TRIM25 can positively regulate the IFN-I signaling activation across RNA viral infections.

### TRIM25 specifically regulates the infection of RNA viruses in duck cells

**Fig 2:**
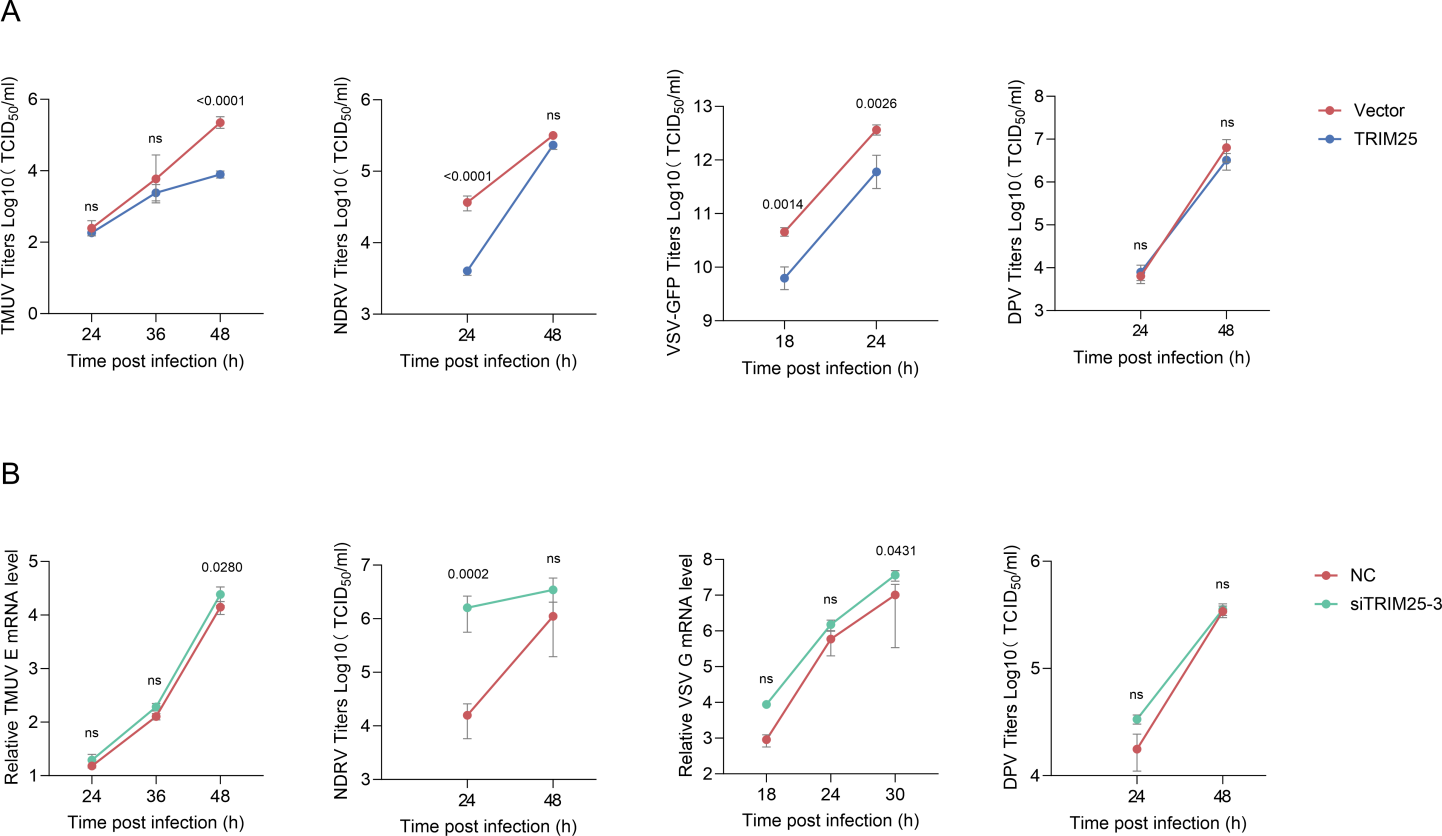
TRIM25 inhibits RNA virus replication. (**A and B**) DEFs were transfected with TRIM25 (**A**) or siTRIM25 (**B**) for 24 h and infected with TMUV (2,000 TCID₅₀), NDRV (1,000 TCID_50_), VSV (0.01 MOI), or DPV (1,000 TCID_50_). Viral RNA levels and titers were determined by RT-qPCR and TCID_50_ assays (mean ± SD, *n* = 3). Statistical analysis was performed using one-way ANOVA with Dunnett’s multiple comparisons test.

To further assess whether duck TRIM25 modulates avian virus infection, TRIM25 was exogenously expressed in DEFs, which were subsequently infected with TMUV, NDRV, VSV, or duck plague virus (DPV, DNA virus) at the indicated time points. Compared to empty vector-transfected controls, TRIM25 overexpression significantly reduced viral proliferation of TMUV, NDRV, and VSV, whereas DPV replication exhibited no statistically significant reduction (Fig. 2A). Complementarily, TRIM25-knockdown cells demonstrated elevated TMUV, NDRV, and VSV viral proliferation relative to the negative control group, while DPV replication remained unaffected (Fig. 2B). Collectively, these data demonstrate that TRIM25 selectively regulates RNA virus infection.

To further elucidate whether duck TRIM25 plays a role in RNA virus-mediated innate immunity, we examined the expression of TRIM25 in DEFs following infection with TMUV, VSV, NDRV, and DHAV-3. The results revealed that both TRIM25 protein expression and mRNA levels were significantly upregulated in DEFs under all viral challenges (Fig. S4A and B). Moreover, infection with TMUV, NDRV, DHAV-3, or VSV at the indicated time points consistently induced the expression of IFN-α, IFN-β, Mx, and OASL (Fig. S4C through F). Notably, in the cases of TMUV and NDRV infection, the mRNA levels of IFN-α exhibited an initial increase followed by a subsequent decline at later time points, whereas the expression of IFN-β and other ISGs (Mx and OASL) remained sustained or continued to rise (Fig. S4C). The results consistent with previous studies in avian systems (27). This phenomenon may be due to IFN-α serving as a rapid, transient first line of defense. The subsequent decline in IFN-α, coupled with the sustained induction of IFN-β, may be attributed to a negative feedback mechanism designed to prevent immunopathology.

### TRIM25 promotes antiviral immune response *in vivo*

**Fig 3:**
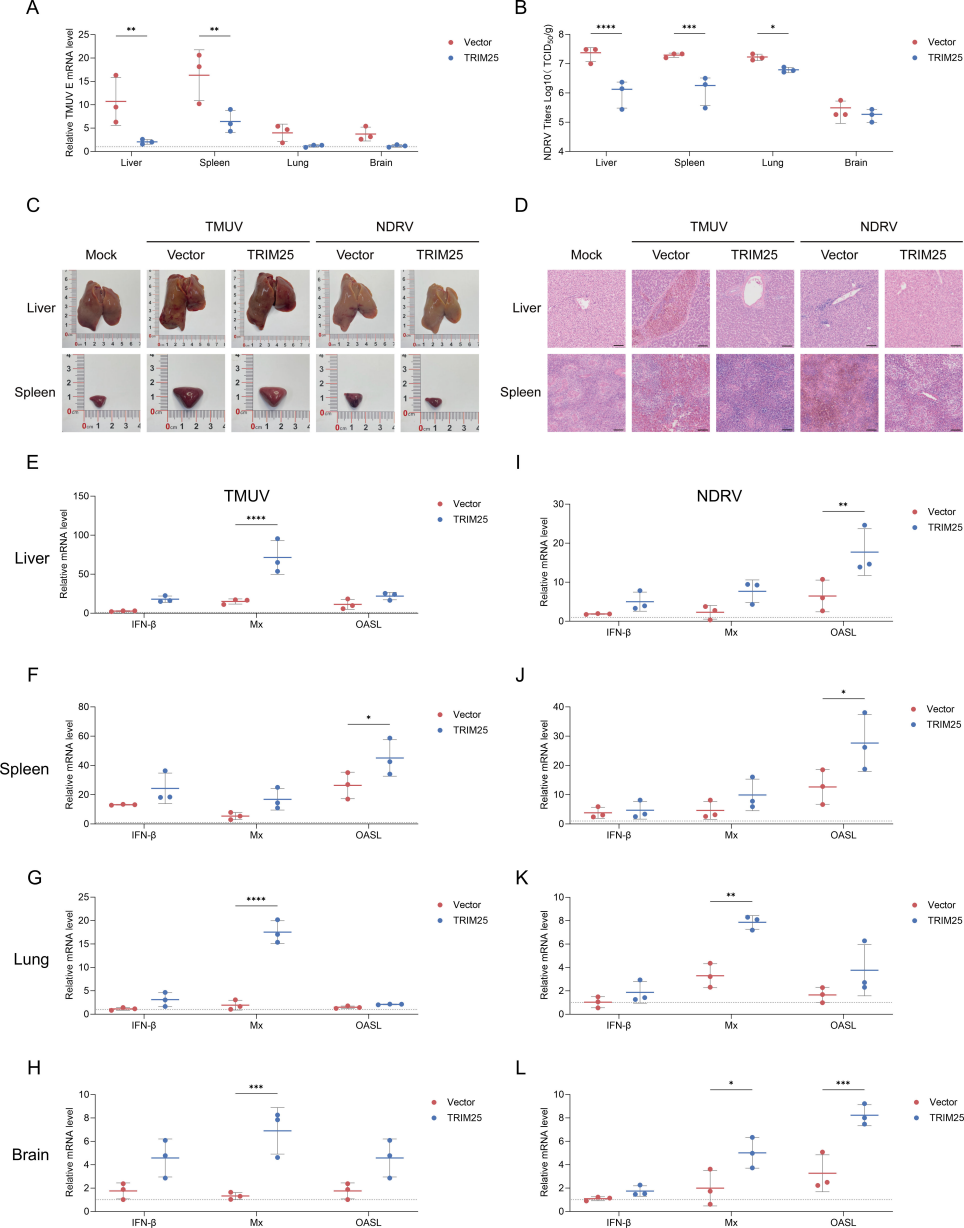
TRIM25 protected the duck from RNA viral infection *in vivo*. (**A and B**) RT-qPCR analysis of TMUV RNA (**A**) and TCID_50_ determination of NDRV viral titers (**B**) in the liver, spleen, lung, and brain of ducks injected with TRIM25 or vector for 3 days post-infection (mean ± SD, *n* = 3). (**C**) Gross pathology of liver and spleen from TRIM25 and vector groups at 3 days post-infection with TMUV or NDRV. The images are from one of five representative ducks. (**D**) Representative H&E-stained images of liver and spleen from TRIM25 and vector groups infected with TMUV or NDRV for 3 days. Scale bar, 100 μm. (**E–H**) RT-qPCR analysis of IFNβ, Mx, and OASL mRNA in TMUV-infected tissues (mean ± SD, *n* = 3). (**I–L**) RT-qPCR analysis of IFNβ, Mx, and OASL mRNA in NDRV-infected tissues (mean ± SD, *n* = 3). Statistical analysis was performed using one-way ANOVA with Dunnett’s multiple comparisons test. *P < 0.05, **P < 0.01, ***P < 0.001, ****P < 0.0001.

To assess the importance of TRIM25 in host defense against viral infection *in vivo*, we upregulated TRIM25 expression in ducks via intravenous injection of polyetherimide (PEI)-packaged plasmids. Exogenous TRIM25 expression was detected in duck lung tissue 2 days post-injection (Fig. S5A). Immunoblotting using an endogenous TRIM25 antibody confirmed elevated total TRIM25 protein levels in the lungs of transfected ducks (Fig. S5B). Following intramuscular TMUV challenge, TRIM25-overexpressing ducks exhibited significantly reduced viral loads in the liver, spleen, lung, and brain tissues compared to vector-control cohorts (Fig. 3A). A similar trend was observed in NDRV-infected ducks, in which TRIM25 overexpression significantly suppressed viral titers across multiple tissues (Fig. 3B). Furthermore, gross pathological examination revealed pronounced hepatic congestion with multifocal petechiae and splenic enlargement in TMUV- and NDRV-infected control groups, whereas TRIM25-overexpressing ducks displayed substantially attenuated tissue lesions (Fig. 3C). Histopathological analysis of H&E-stained sections demonstrated attenuated tissue pathology in TRIM25-overexpressing groups when compared to TMUV and NDRV-infected controls. Infected control groups displayed severe dilation of central veins with accumulated erythrocytes, prominent inflammatory cell infiltration throughout hepatic parenchyma, and complete dissolution of normal splenic microstructure (Fig. 3D). In agreement with these protective effects, transcriptional analysis revealed significant upregulation of IFN-β, Mx, and OASL in the liver, spleen, lung, and brain of TRIM25-overexpressing ducks following TMUV and NDRV infection (Fig. 3E through L). Notably, after a 7-day challenge with DHAV-3, TRIM25-overexpressing ducks exhibited significantly higher survival rates relative to vector controls (Fig. S5C), accompanied by reduced viral loads in major organs (Fig. S5D). Furthermore, Gross and histopathological assessments further confirmed milder lesions in the TRIM25-overexpressing group (Fig. S5E and F). Collectively, these results demonstrate that TRIM25 confers robust protection against RNA viral infections *in vivo* by enhancing host antiviral immunity.

### TRIM25 enhances IFNs induction by interacting with IRF7

**Fig 4:**
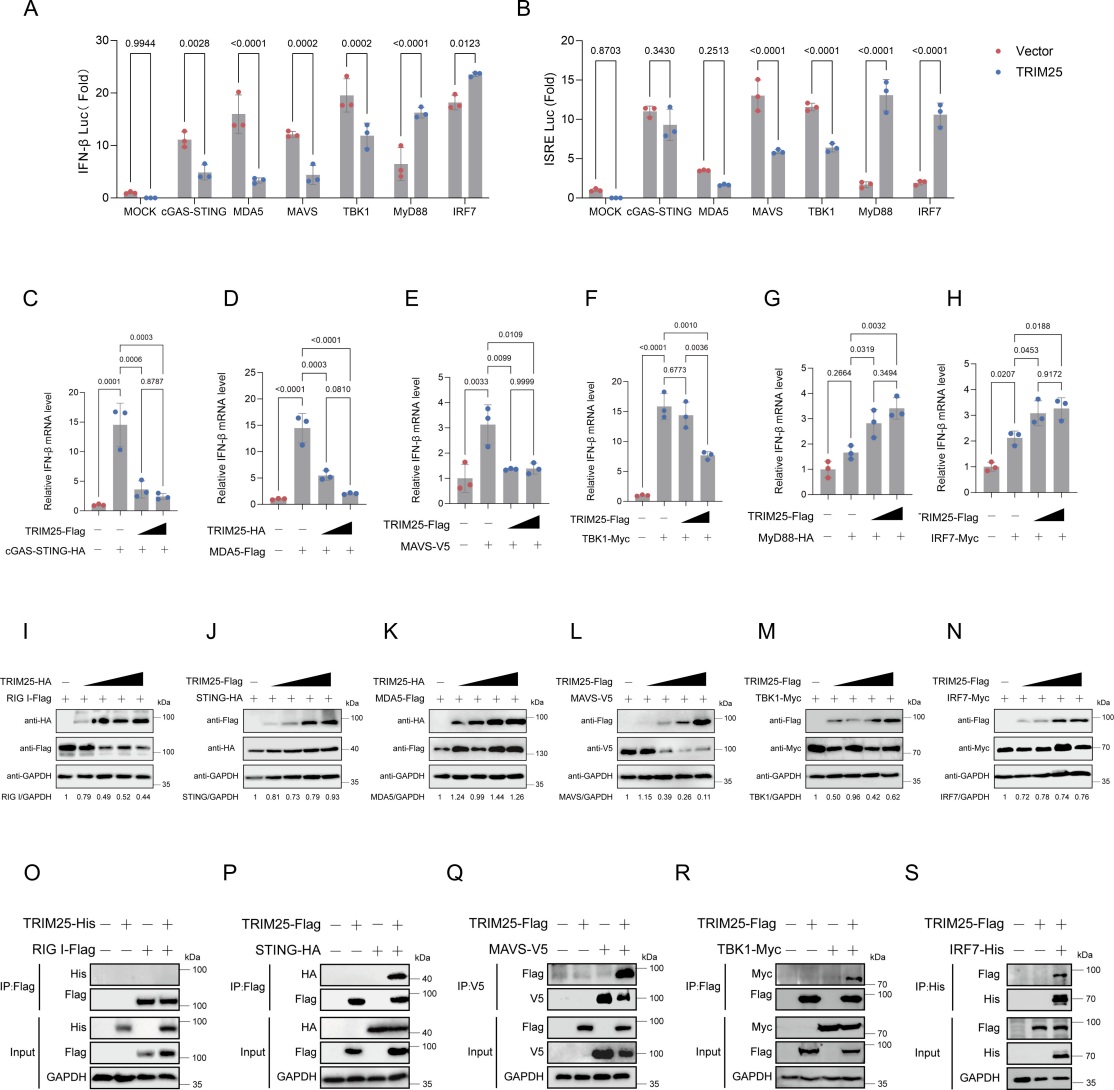
TRIM25 interferes with IFNβ production via targeting IRF7. (**A and B**) Dual-luciferase assays in DEFs co-transfected with reporter, adaptor, and TRIM25 plasmids at 36 h post-transfection. Luciferase activity was normalized to *Renilla* (mean ± SD, *n* = 3). Statistical analysis was performed using one-way ANOVA with Dunnett’s multiple comparisons test. (**C–H**) RT-qPCR detection of IFNβ mRNA in DEFs co-transfected with adaptor and increasing TRIM25 plasmids at 36 h post-transfection (mean ± SD, *n* = 3). Statistical analysis was performed using one-way ANOVA with Dunnett’s multiple comparisons test. (**I–N**) Western blot analysis of adaptor protein expression in HEK293T cells. Cells were co-transfected with adaptor plasmids as indicated, together with the empty vector or increasing amounts of TRIM25 expression plasmids. The relative expression of the indicated adaptor protein was quantified by ImageJ software. (**O–S**) Co-IP assays detecting interactions between TRIM25 and adaptor proteins in HEK293T cells. Cells were co-transfected with TRIM25-Flag and adaptor expression plasmids or an empty vector for 36 h and harvested for Co-IP using the specified primary antibodies.

Next, to elucidate the molecular mechanism underlying TRIM25-mediated enhancement of IFN induction, we investigated the specific level within the multiple receptor-mediated signaling pathway at which TRIM25 exerts its function. We assessed the effect of TRIM25 overexpression on both IFN-β and interferon-sensitive response element (ISRE) reporter activity by stimulating the pathway with limiting amounts of different factors involved in this signaling cascade. TRIM25 overexpression markedly suppressed cGAS/STING-, MDA5-, MAVS-, and TBK1-mediated IFN-β reporter activation, yet significantly enhanced myeloid differentiation primary response gene 88 (MyD88)- and IRF7-mediated reporter activity (Fig. 4A and B). Besides, Co-IP results revealed that MyD88, indeed, interacts with IRF7, and notably, the presence of TRIM25 significantly increased the amount of IRF7 protein recruited to the MyD88 complex, demonstrating that TRIM25 promotes the formation and stability of the MyD88-IRF7 signaling complex (Fig. S6A). Together, these results suggest that TRIM25 functions as a critical facilitator at the level of the MyD88-IRF7 complex and that duck innate immune signaling may preferentially operate through a MyD88-IRF7-IFN-β axis distinct from mammalian systems.

To further validate this finding, we performed dose-dependent overexpression of TRIM25 and examined its impact on innate immunity. Consistent with the results from the dual-luciferase reporter assay, TRIM25 dose-dependently enhanced MyD88- and IRF7-mediated IFN-β mRNA transcription, while attenuating signaling driven by cGAS/STING, MDA5, MAVS, and TBK1 (Fig. 4C through H). We next examined the association of TRIM25 with key signaling factors by western blotting. Interestingly, TRIM25 reduced MAVS expression in a dose-dependent manner, while having no detectable effect on the expression of other proteins (Fig. 4I through N). Moreover, Co-IP assays demonstrated that duTRIM25 physically associates with several innate immune signaling proteins, including STING, MAVS, and TBK1, but not with RIG-I (Fig. 4O through S). Notably, TRIM25 did not affect the interaction between TBK1 and IRF7 (Fig. S6B). Collectively, these findings demonstrate that TRIM25 specifically targets IRF7, thereby modulating IRF7-dependent signaling and ultimately regulating the innate immune response.

### TRIM25 mediates K27-linked ubiquitination of IRF7

**Fig 5:**
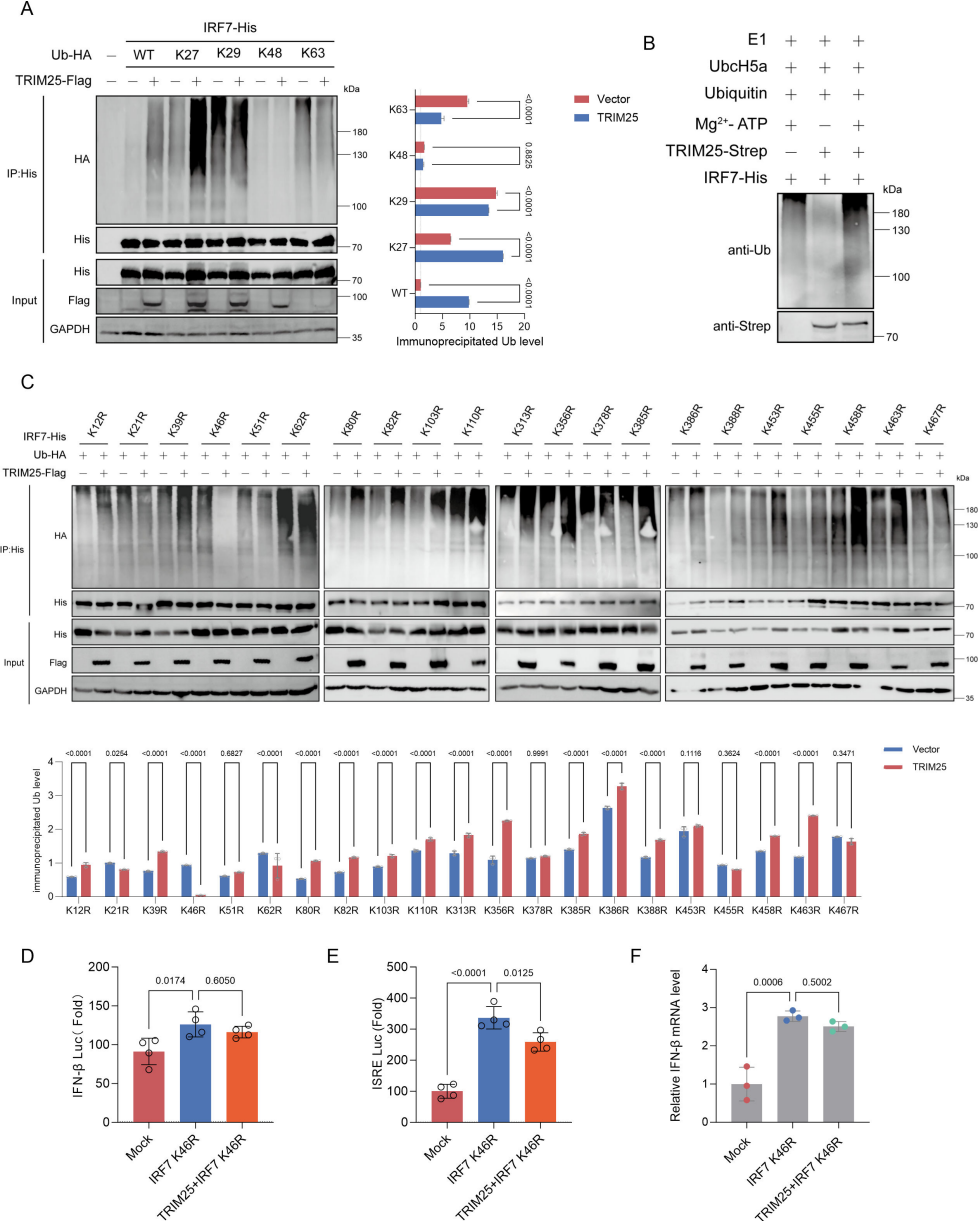
TRIM25 potentiates K27-linked ubiquitination of IRF7 at residue K46. (**A**) Co-IP assays of HEK293T cells to detect the interaction between IRF7 and different types of ubiquitin. Cells were co-transfected with HA-tagged ubiquitin (WT, K27, K29, K48, or K63), IRF7-His, and TRIM25-Flag expression plasmids or an empty vector for 36 h, followed by Co-IP using an anti-His primary antibody. (**B**) *In vitro* ubiquitination assay was performed in the presence of E1, UbCH5a, Ub, TRIM25, and IRF7. (**C**) Co-IP assays of HEK293T cells to detect the interaction between IRF7 and ubiquitin. Cells were co-transfected with HA-Ub, IRF7-His or its lysine mutants, and TRIM25-Flag expression plasmids or an empty vector for 36 h, followed by Co-IP using an anti-His primary antibody. (**D–F**) Dual-luciferase assay (**D, E**) and RT-qPCR detection (**F**) of DEFs co-transfected with IRF7 K46R and TRIM25 or vector plasmids at 36 h post-transfection. Luciferase activity was normalized to *Renilla* (mean ± SD, *n* = 4). Statistical analysis was performed using one-way ANOVA with Dunnett’s multiple comparisons test.

To investigate the molecular mechanism by which TRIM25 regulates IRF7, we first examined whether TRIM25 functions as an E3 ubiquitin ligase for IRF7. TRIM25 contains a RING-finger domain, suggesting a potential role in catalyzing ubiquitination. Indeed, TRIM25 promoted IRF7 polyubiquitination only in the presence of HA-ubiquitin WT or the K27 mutant, indicating that TRIM25 mediates K27-linked polyubiquitination of IRF7 (Fig. 5A). To determine whether this ubiquitination is directly catalyzed by TRIM25, we next performed classical *in vitro* ubiquitination assays using purified recombinant proteins. Under these conditions, TRIM25 markedly enhanced IRF7 ubiquitination, whereas reactions lacking TRIM25 showed minimal ubiquitination signals. These results demonstrate that TRIM25 directly catalyzes IRF7 ubiquitination independent of other cellular factors, thereby establishing TRIM25 as an E3 ubiquitin ligase for IRF7 (Fig. 5B).

To further identify the ubiquitination sites, we mutated all lysine residues in IRF7 to arginine (Fig. 5B). Co-immunoprecipitation assays demonstrated that TRIM25 failed to promote ubiquitination of the IRF7 K21R, K46R, K51R, K378R, and K467R mutants, among which the loss of ubiquitination was most pronounced in K46R (Fig. S7). Moreover, the IRF7 K46R mutant remained capable of inducing IFN-I signaling activation, whereas TRIM25 failed to significantly enhance the IFN-I signaling response mediated by this mutant (Fig. 5D through F). These results identify K46 as the principal ubiquitin acceptor site in IRF7 targeted by TRIM25 and suggest that K27-linked ubiquitination at this site is critical for IRF7 activation.

### TRIM25 enhances IFN production by inhibiting IRF7 degradation independently of its E3 ubiquitin ligase activity

**Fig 6:**
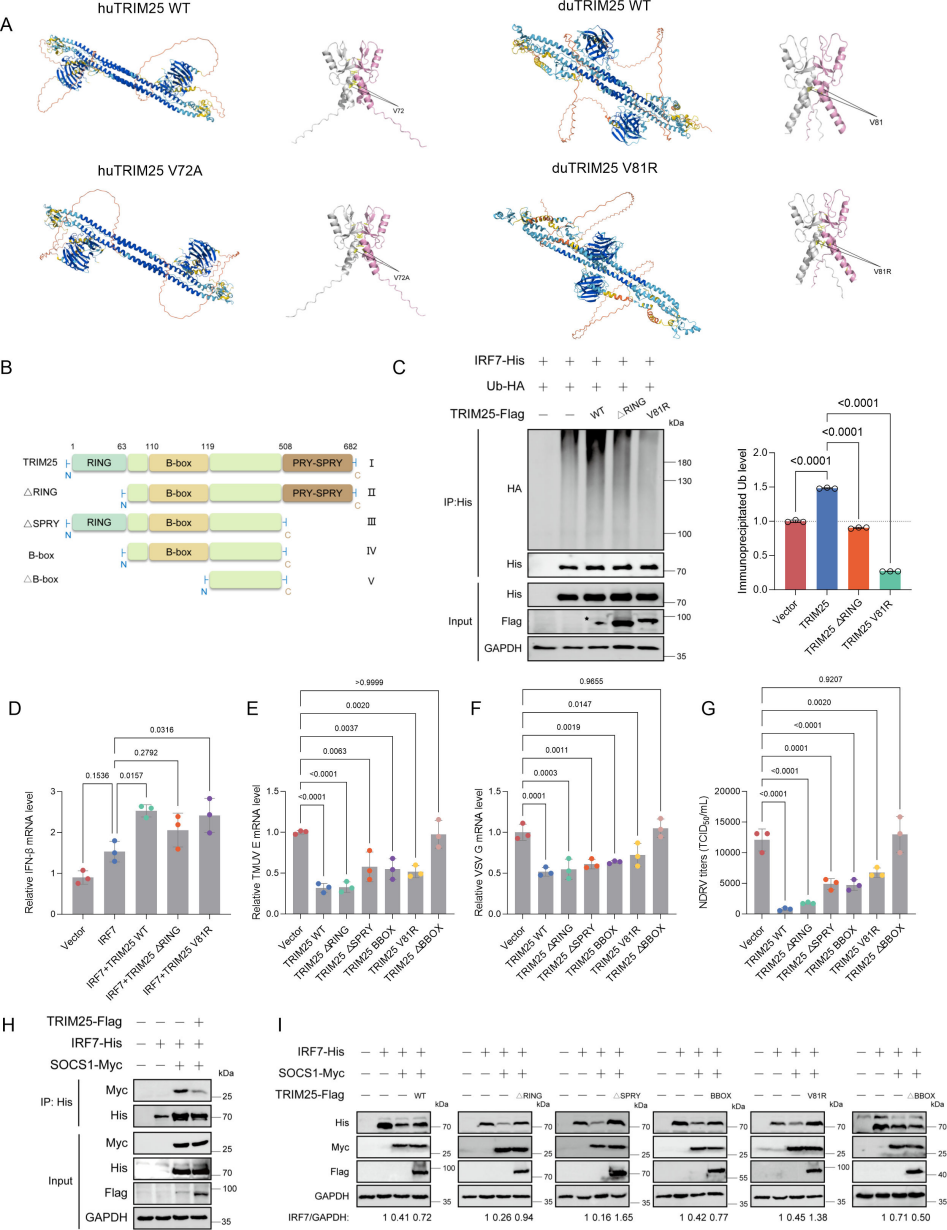
TRIM25 inhibits IRF7 degradation independently of its E3 ubiquitin ligase activity. (**A**) Predicted dimer structures of human TRIM25 (WT/V72A) and duck TRIM25 (WT/V81R) generated by AlphaFold 3. The locations of residues V72 and V81 are shown as sticks with labels. (**B**) Schematic diagram of TRIM25 truncations. (**C**) Co-IP assays of HEK293T cells to detect the interaction between IRF7 and ubiquitin. Cells were co-transfected with HA-Ub, IRF7-His, and TRIM25-Flag or its mutants, or an empty vector for 36 h, followed by Co-IP using an anti-His primary antibody. (**D**) RT-qPCR detection of IFNβ mRNA in DEFs co-transfected with IRF7 and TRIM25 mutants at 36 h post-transfection (mean ± SD, *n* = 3). Statistical analysis was performed using one-way ANOVA with Dunnett’s multiple comparisons test. (**E–G**) RT-qPCR quantification of viral RNA and titers in DEFs expressing TRIM25 mutants after TMUV (2,000 TCID_50_ [**E**]), VSV (0.01 MOI [**F**]), or NDRV (2,000 TCID_50_ [**G**]) infection (mean ± SD, *n* = 3). Statistical analysis was performed using one-way ANOVA with Dunnett’s multiple comparisons test. (**H**) Co-IP assays of HEK293T cells to detect the interaction between IRF7 and SOCS1. Cells were co-transfected with IRF7-His, SOCS1-Myc, and TRIM25-Flag or its mutants, or an empty vector for 36 h, followed by Co-IP using an anti-His primary antibody. (**I**) Western blot analysis of HEK293T cells co-transfected with IRF7-His, SOCS1-Myc, and either an empty vector or TRIM25 mutants using the indicated antibodies.

Human TRIM25 residue V72 is located at the dimerization interface of the RING domain and serves as a critical site for stabilizing dimer formation (28). Mutation of this residue disrupts RING domain dimerization, thereby impairing the ability of TRIM25 to efficiently catalyze ubiquitination and compromising its function in the RIG-I signaling pathway (28). Structural prediction by AlphaFold3 indicated that mutation of the corresponding residue V81 in duck TRIM25 also interferes with dimer formation (Fig. 6A). Consistent with this prediction, we found that both the RING-deleted mutant of duck TRIM25 (TRIM25 ΔRING) and the TRIM25 V81R mutant exhibited markedly reduced capacity to induce IRF7 ubiquitination (Fig. 6C). Despite their impaired ubiquitination activity, overexpression of TRIM25 ΔRING or V81R significantly enhanced IRF7-induced IFN-β mRNA expression, comparable to that observed with wild-type TRIM25, indicating that TRIM25 can potentiate IRF7-dependent IFN-I production through an E3 ligase-independent mechanism (Fig. 6D). Interestingly, we further investigated the pivotal domain through which TRIM25 exerts its antiviral activity despite its impaired ubiquitination activity; TRIM25 WT, ΔRING, ΔSPRY, BBOX, and V81R significantly suppressed RNA virus replication, while TRIM25 ΔBBOX lost its ability to suppress RNA virus replication (Fig. 6E through G). These results demonstrate that the BBOX domain is indispensable for TRIM25-mediated antiviral activity, whereas the RING and SPRY domains are dispensable for this function.

To further explore the mechanism underlying the antiviral activity of these TRIM25 mutants, we investigated their potential role in regulating SOCS1, a negative regulator of innate immunity that we previously demonstrated to degrade IRF7 and suppress IFN-β production (29). Co-immunoprecipitation assays revealed that TRIM25 inhibited the interaction between SOCS1 and IRF7 and that TRIM25 WT, ΔRING, ΔSPRY, BBOX, and V81R reduced SOCS1-mediated degradation of IRF7, while TRIM25 ΔBBOX failed to antagonize SOCS1-IRF7 interaction and was unable to prevent SOCS1-mediated degradation of IRF7, indicating that the BBOX region is the primary domain through which TRIM25 antagonizes SOCS1-mediated degradation of IRF7 (Fig. 6H and I). Together, these findings indicate that TRIM25 promotes IFN production and restricts RNA virus replication through both its E3 ubiquitin ligase-dependent and -independent activities.

### TRIM25 stabilizes IRF7 expression during RNA virus infection

**Fig 7:**
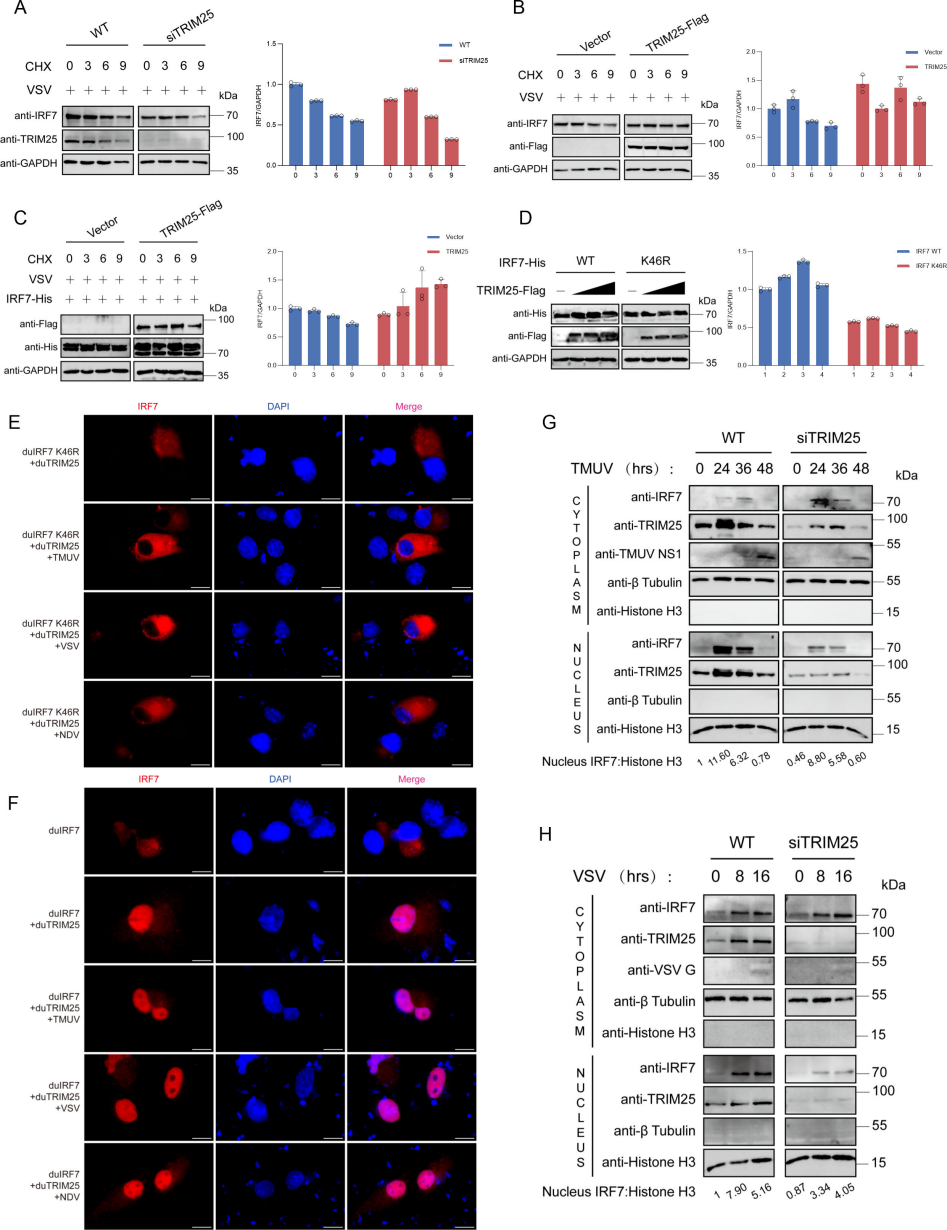
TRIM25 stabilizes IRF7 expression, promoting its translocation and aggregation in the nucleus. (**A and B**) Western blot analysis of DEFs to detect IRF7 expression. DEFs transfected with siTRIM25 (**A**) or TRIM25 (**B**) were infected with VSV and treated with 100 µg/mL CHX for the indicated times. The relative IRF7 expression was quantified by ImageJ. (**C**) Western blot analysis of HEK293T to detect IRF7 expression. Cells transfected with IRF7-His with an empty vector or TRIM25-Flag were infected with VSV and treated with 100 µg/mL CHX for the indicated times. The relative IRF7 expression was quantified by ImageJ. (**D**) Western blot analysis of HEK293T to detect IRF7 expression. Cells transfected with IRF7-His along with an empty vector or increasing amounts of TRIM25 were treated with 100 µg/mL CHX for the indicated times. The relative IRF7 expression was quantified by ImageJ. (**E**) Immunofluorescence analysis of BHK21 cells co-expressing IRF7 K46R-His and TRIM25 infected with TMUV, VSV, or NDV. Scale bars, 10 µm. (**F**) Immunofluorescence analysis of BHK21 cells co-expressing IRF7-His with vector or TRIM25 after TMUV, VSV, or NDV infection. Scale bars, 10 µm. (**G and H**) Western blot analysis of DEFs to detect IRF7 localization. Wild-type and TRIM25-knockdown DEFs were infected with TMUV(F) or VSV(G) and subjected to cytoplasmic and nuclear protein fractionation at the indicated times post-infection.

To further characterize the role of TRIM25 in regulating innate immune signaling, we analyzed its impact on IRF7 protein stability. Time-course experiments in wild-type and TRIM25-knockdown DEFs revealed significant differences in IRF7 protein stability upon VSV infection, suggesting that TRIM25 influences early steps in IRF7-dependent gene expression (Fig. 7A). Moreover, TRIM25 overexpression stabilized endogenous IRF7 expression following VSV infection (Fig. 7B).

To further verify the specificity of this effect, we co-transfected duTRIM25-Flag and duIRF7-His into human embryonic kidney (HEK) 293T cells and subsequently infected them with VSV. Time-course experiments demonstrated that TRIM25 specifically stabilized IRF7 protein levels, whereas it failed to stabilize the IRF7 K46 mutant (Fig. 7C and D). Indirect immunofluorescence showed that infection with TMUV, NDV, or VSV effectively promoted the nuclear translocation of wild-type IRF7, but not the K46R mutant (Fig. 7E and F). Altogether, these findings indicate that TRIM25 promotes IFN production by catalyzing K27-linked polyubiquitination of IRF7 at residue K46 and by stabilizing IRF7 expression, thereby ensuring efficient activation of innate antiviral responses.

### TRIM25 promotes IRF7 translocation and aggregation in the nucleus

Next, we performed cellular fractionation to examine IRF7 intracellular localization at different times post-infection. In TMUV-infected cells, cytoplasmic IRF7 levels progressively declined while nuclear IRF7 increased, peaking at 24 hpi before subsequently decreasing (Fig. 7G). A similar pattern was observed following VSV infection, with nuclear accumulation of IRF7 peaking at 16 hpi (Fig. 7H). Importantly, TRIM25 knockdown markedly impaired IRF7 nuclear translocation during both TMUV and VSV infections (Fig. 7G and H).

Consistent with these findings, immunofluorescence analysis revealed that IRF7 predominantly localized to the cytoplasm under basal conditions. TRIM25 overexpression induced robust nuclear translocation of IRF7, which was further enhanced by RNA virus infection (TMUV, NDV, and VSV) (Fig. 7F).

Together, these results demonstrate that TRIM25 facilitates IRF7 nuclear translocation in response to RNA virus infection, thereby promoting efficient activation of antiviral signaling.

## DISCUSSION

Ubiquitination-mediated post-translational modification of proteins exerts multifaceted regulatory functions that extend beyond the classical proteasome-dependent degradation pathway. Notably, non-proteolytic ubiquitination has emerged as a critical mechanism governing diverse cellular processes, including membrane trafficking, DNA damage repair, cell cycle progression, and innate immune signaling (30). In mammalian systems, TRIM25 has been well characterized as a pivotal E3 ubiquitin ligase that catalyzes K63-linked ubiquitination of RIG-I, thereby facilitating its interaction with MAVS and potentiating antiviral signaling transduction (31). Intriguingly, our current investigation reveals a distinct regulatory paradigm in avian species. Through comprehensive biochemical analyses, we demonstrate that duck TRIM25 positively modulates IFN-I signaling through an evolutionarily divergent mechanism. Unlike its mammalian counterpart that directly targets RIG-I, duck TRIM25 specifically interacts with and ubiquitinates IRF7, a master transcription factor governing IFN-I production. This species-specific substrate selection induces nuclear transduction rather than proteasomal degradation of IRF7, thereby enhancing its transcriptional activity and subsequent antiviral response. Besides, our results show that duck TRIM25 promotes the MyD88-IRF7 axis while suppressing cGAS/STING and MDA5 pathways. Such bidirectional regulation suggests that TRIM25 does not function as a simple on/off switch, but rather as a rheostat that fine-tunes the magnitude and specificity of the antiviral response. This “gatekeeper” function may be physiologically significant in preventing the deleterious effects of hyper-inflammation. By prioritizing certain signaling branches tailored to the invading pathogen while dampening others, TRIM25 could help the host achieve an optimal balance between efficient viral clearance and the prevention of immunopathology, a strategy that is likely essential for the evolutionary fitness of avian species in the face of diverse RNA and DNA virus challenges.

The tripartite motif protein TRIM25 is defined by a conserved N-terminal domain architecture comprising a catalytic RING domain, one or two B-box domains, and a coiled-coil dimerization domain, followed by a C-terminal SPRY domain (32). Structural analyses reveal that the coiled-coil domain forms a stable antiparallel dimer, positioning the RING domains at opposite ends of the molecule (~170 Å apart) (33). The RING domain dimerizes with E2 ubiquitin-conjugating enzymes, a configuration essential for its catalytic activity. Disruption of this dimer interface—such as through L69A or V72A mutations in human TRIM25—markedly impairs ubiquitin ligase function (28). In this study, sequence alignment identified the human V72 residue as V81 in ducks. Functional assays revealed that the V81A mutation significantly reduced the ability of duck TRIM25 to ubiquitinate IRF7, while not diminishing its capacity to promote IFN-I production and antiviral effector expression. Notably, this residue is conserved across humans, mice, chickens, cattle, and pigs, suggesting a potential conserved mechanism for TRIM25’s E3 ligase activity (Fig. S8A). The site (K46) at which duck TRIM25 ubiquitinates IRF7 is also conserved across different species; however, the interaction between them has not been reported in other species (Fig. S8B). However, proteins exhibit diversity during the process of evolution. These findings underscore TRIM25’s evolutionary conservation as a ubiquitination regulator while highlighting context-dependent adaptations that tailor immune responses across species.

Human TRIM25 is a well-characterized E3 ubiquitin ligase that targets multiple components of the innate immune system, including RIG-I, MAVS, ISG15, TRAF6, and p53. Among these, its role in RIG-I activation has been most extensively characterized. Structural and functional analyses demonstrate that human TRIM25 catalyzes K63-linked polyubiquitination of RIG-I at lysine residues K99, K169, K172, K181, K190, and K193, with K172 being critical for stabilizing the RIG-I-MAVS interaction. Notably, the K172R mutation abolishes TRIM25-mediated ubiquitination and subsequent MAVS signaling (25). Intriguingly, we observed that duck TRIM25 fails to ubiquitinate RIG-I or induce IFN-I production in DEFs. Sequence alignment revealed that the K172 residue, essential in humans, is not conserved in duck RIG-I (26). However, in avian DF-1 cells, duck TRIM25 promotes RIG-I ubiquitination at distinct lysine residues (K167 and K193) and activates downstream antiviral signaling, suggesting species-specific ubiquitination patterns (26). This dichotomy implies that duck TRIM25 engages RIG-I through mechanisms divergent from its human counterpart, potentially involving alternative structural interfaces or co-factor recruitment. These findings highlight evolutionary plasticity in TRIM25-RIG-I interactions, where conserved E3 ligase activity is repurposed across species via substrate adaptation, with implications for tailoring antiviral responses in diverse hosts.

Compared with other vertebrates, avian genomes are markedly smaller and less repetitive, with transposable and repetitive elements accounting for only 4%–10% of the genome, in contrast to 34%–52% in mammals and reptiles (34). This extensive genome compaction is hypothesized to reflect strong evolutionary selection for genome streamlining and reduced metabolic cost to support flight (35). A striking consequence of this evolution is the complete absence of the IRF3 gene in the vast majority of poultry within the *Neognathae* clade, including *Galliformes* (e.g., chicken, turkey, quail) and *Anseriformes* (e.g., duck, goose), due to a loss event in their common ancestor. This evolutionary gene loss is characterized by an empty syntenic locus with no pseudogene or residual fragment remaining (36). In contrast, basal birds of the *Palaeognathae* clade, such as the ostrich (*Struthio camelus*) and emu (*Dromaius novaehollandiae*), have retained the IRF3 gene (5). Notably, while IRF3 is also missing in some teleost fish and completely absent in invertebrates, its specific loss in modern birds may represent an adaptation for co-evolution and coexistence with RNA viruses, whereby excessive inflammatory responses are attenuated to improve host survival.

The extensive genome compaction has driven the evolution of unique immune strategies, potentially conferring higher “functional saturation” on the remaining immune-related genes. Within this streamlined genomic context, avian species have adapted their innate immune networks to maintain robust antiviral defenses, with IRF7 functionally compensating for the absence of IRF3 as a master transcriptional regulator. While IRF3 and IRF7 serve as master transcriptional regulators across multiple innate immune pathways, their regulation by E3 ubiquitin ligases has been extensively studied, primarily focusing on proteolytic degradation (37–40). However, the mechanisms by which non-proteolytic ubiquitination activates these transcription factors remain poorly understood. Neuralized E3 ubiquitin-protein ligase 3 (NEURL3) directly interacts with IRF7, catalyzing its ubiquitination at K375 to enhance transcriptional activation of ISGs (41). In contrast, IRF3 activation occurs through a scaffold-mediated mechanism in which dual-specificity tyrosine phosphorylation-regulated kinase 4 (DYRK4) recruits TRIM71 and the linear ubiquitin chain assembly complex (LUBAC) to promote linear (M1-linked) ubiquitination, stabilizing IRF3 and amplifying its transcriptional activity during viral infection (42). Despite these advances, the role of non-proteolytic ubiquitination in avian species IRF7 activation—particularly in ducks, where IRF7 compensates for the absence of IRF3—remains unexplored. Within this streamlined genomic context, avian species have adapted their innate immune networks to maintain robust antiviral defense. Elucidating whether duck IRF7 employs analogous ubiquitin-dependent mechanisms (e.g., K63/M1-linked chains) or evolves unique regulatory strategies could uncover species-specific adaptations that optimize antiviral responses in waterfowl, offering insights into the evolutionary plasticity of interferon signaling.

**Fig 8:**
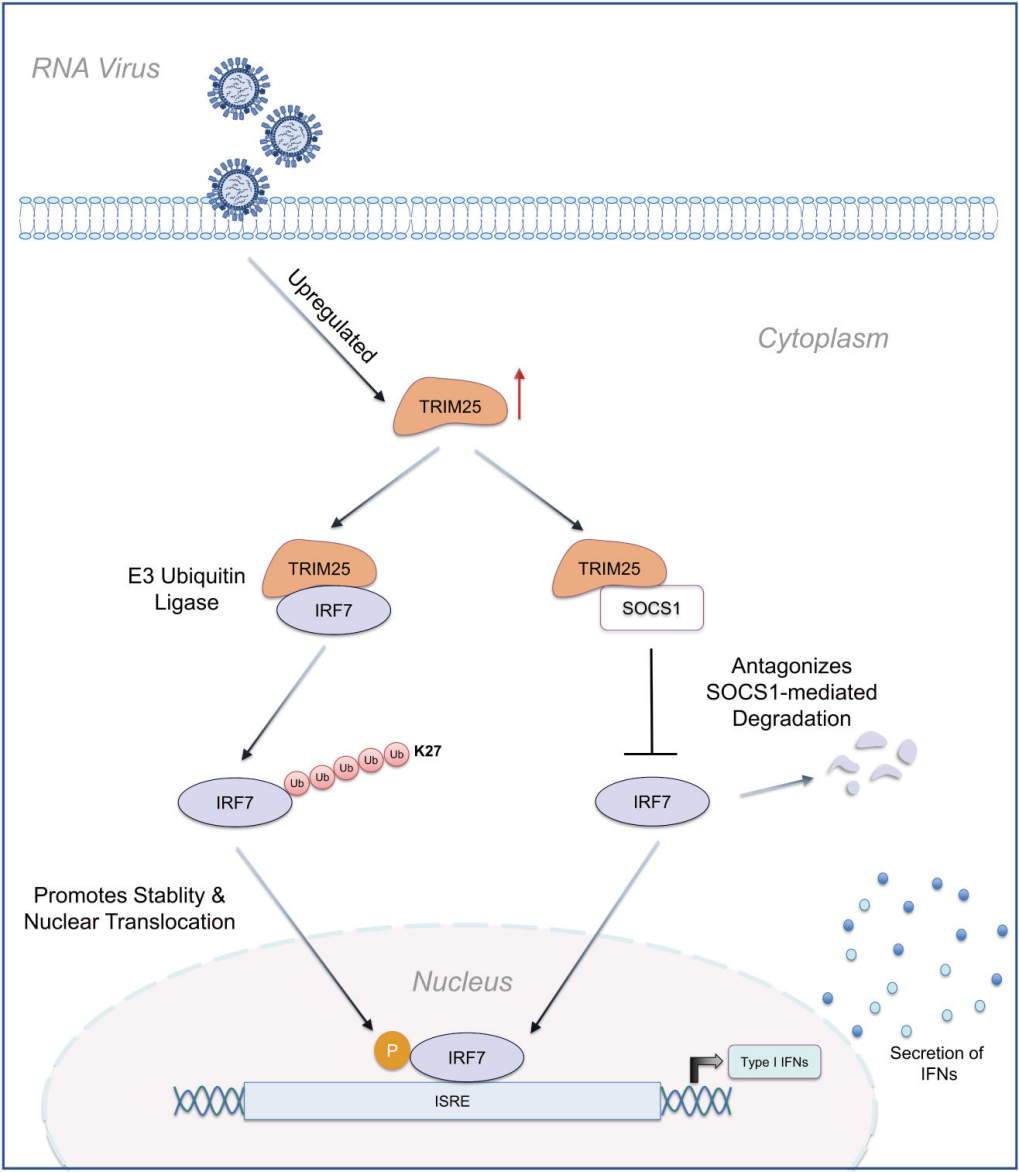
Molecular mechanism of TRIM25-mediated enhancement of IFN induction. Model of the proposed mechanism by which TRIM25 enhances IFNβ and ISG production upon viral infection. Upon RNA virus sensing, TRIM25 is activated and stabilizes IRF7 by promoting K27-linked ubiquitination and inhibiting SOCS1-mediated degradation, subsequently leading to IRF7 phosphorylation and nuclear translocation. This process triggers the IFNβ signaling pathway, further suppressing RNA viral replication.

Our findings demonstrate that duck TRIM25 plays a pivotal role in activating the IFN-I innate immune pathway by directly interacting with IRF7 and catalyzing its non-proteolytic K27-linked ubiquitination. This post-translational modification enhances IRF7’s nuclear translocation capacity, facilitating its binding to ISREs and amplifying IFN-I production. Crucially, this study provides the first evidence of a TRIM family protein regulating the nuclear trafficking of a key IRF transcription factor, expanding the known functional repertoire of TRIM E3 ligases beyond classical proteasomal degradation or signaling scaffold roles. The K27 ubiquitination-dependent mechanism—distinct from the K48/K63-linked chains typically associated with IRF regulation—suggests evolutionary innovation in waterfowl to optimize antiviral responses while avoiding immunopathology (Fig. 8). These results establish TRIM25 as a master regulator of IRF7 activation in avian species and highlight ubiquitination topology as one of the critical determinants of interferon signaling specificity across species.

## MATERIALS AND METHODS

### Cells and viruses

DEFs, DF-1, and HEK293T cells were cultured in Dulbecco’s modified Eagle medium (DMEM) (1210064, Gibco, USA) supplemented with 10% fetal bovine serum (FBS) (16010159, Gibco, USA) at 37°C with 5% CO2. The TMUV CQW1 strain (GenBank: KM233707.1), DPV-CHv, NDRV, and VSV were provided by the Institute of Preventive Veterinary Medicine at Sichuan Agricultural University.

### Plasmids

Expression plasmids pCAGGS-duSTING-HA, pCAGGS-duMDA5-Flag, pCAGGS-duRIG-I-Flag, pCAGGS-duMAVS-V5, pCAGGS-duTBK1-Myc, pCAGGS-duMyD88-HA, and pCAGGS-duIRF7-Myc were provided by the Institute of Preventive Veterinary Medicine, Sichuan Agricultural University. The primer for amplification of duTRIM25 was designed according to the sequence of duTRIM25 (NCBI accession no. KY974316.1). The cDNA synthesized from total RNA extracted from DEFs served as the PCR template. The amplified duTRIM25 fragment was first cloned into the pMD19-T vector (pMD19-T Vector Cloning Kit; Takara, Japan) and subsequently subcloned into the pcDNA3.1 expression vector using EcoRI and XhoI (New England Biolabs, China) restriction sites with primers pcDNA3.1-duTRIM25-F and pcDNA3.1-duTRIM25-R. DuTRIM25-Flag plasmid and other TRIM25 domain deletion mutants were constructed by the same method. The recombinant plasmids were then transformed into DH5α chemically competent *Escherichia coli* and confirmed by sequencing (Sangon Biotech, China). The relevant primer sequences are listed in Table S1.

### Antibodies and reagents

Anti-Flag-tag antibody (10543-1-AP) and anti-Myc-tag (16286-1-AP) were purchased from Proteintech (China). Anti-His-tag antibody (AE086), anti-tubulin-tag antibody (AC008), and anti-V5-tag antibody (AE089) were purchased from Abclonal (China). Anti-HA-tag (16286-1-AP) was purchased from Smart-Lifesciences (China). Anti-GAPDH-tag (A02367-100) was purchased from Genscript (China). The anti-TMUV NS1 monoclonal antibody and the anti-duIRF7 polyclonal antibody were prepared in our laboratory, while the anti-duTRIM25 monoclonal antibody was kindly provided by Professor Rui Luo from Huazhong Agricultural University, Wuhan, China. Horseradish peroxidase (HRP)-conjugated anti-rabbit immunoglobulin (IgG) antibody (1 706 515) and HRP-conjugated anti-mouse IgG antibody (1 706 516) were purchased from Bio-Rad (USA). (R)-MG132 (HY-13259C) was purchased from MedChemExpress (MCE, China).

### Nuclear and cytoplasmic protein extraction

VSV-/TMUV-infected or not DEFs (WT and siTRIM25) were fractionated on cytoplasmic, nuclear fractions using the Nuclear and Cytoplasmic Protein Extraction Kit according to the manufacturer’s instructions (Beyotime). Fractions were analyzed by immunoblotting with the antibodies indicated in each figure.

### Co-immunoprecipitation and immunoblotting

HEK293T or DEFs were transfected with the indicated plasmids using PlusTrans (Nulen, CT801). At 36 h post-transfection, cells were washed twice with ice-cold PBS and lysed in IP Lysis Buffer (Thermo Fisher Scientific, 87787) supplemented with protease inhibitor cocktail (MCE, HY-K0010). Lysates were clarified by centrifugation at 12,000 × *g* for 10 min at 4°C. For immunoprecipitation, clarified lysates were incubated with specified antibodies overnight at 4°C with rotation, followed by incubation with Protein A/G Magnetic Beads (Bio-Rad, 1614023) for 2–4 h. Beads were washed three times with cold TBST (Tris-buffered saline with 0.1% Tween-20) and resuspended in 2× Laemmli buffer. Proteins were denatured at 95°C for 10 min, separated by SDS-PAGE, and transferred to polyvinylidene difluoride (PVDF) membranes (Bio-Rad, 1620177) using a Bio-Rad semidry transfer system. Membranes were blocked in 5% non-fat milk in TBST for 2–3 h at room temperature and then incubated overnight at 4°C with specific primary antibodies. After three washes with TBST, membranes were incubated with HRP-conjugated secondary antibodies (1:3,000) for 1–2 h at room temperature. Signals were detected using Clarity Western ECL Substrate (Bio-Rad, 1705060) in a Bio-Rad imager. Bands were analyzed with ImageJ software.

### Fluorescence microscopy

BHK-21 cells were seeded onto glass coverslips in 12-well plates and transfected with specified plasmids at approximately 50% confluency. Post-transfection, cells were washed thrice with PBS, fixed with 4% paraformaldehyde in PBS for 1 h at 4°C, permeabilized with 0.3% Triton X-100 in PBS at 4°C for 1 h, and blocked with 5% bovine serum albumin (BSA) in PBS for 1 h at 37°C. Cells were incubated with primary antibodies at 4°C overnight. After three washes with PBS-Tween (PBST), samples were stained with Alexa Fluor-conjugated secondary antibodies (1:1,000) for 1 h at 37°C, followed by nuclear counterstaining with 4′,6-diamidino-2-phenylindole (DAPI) for 10 min at room temperature. Finally, the cells were observed and photographed using a fluorescence microscope (Nikon, Japan).

### RNAi

SiRNAs targeting duTRIM25 were designed by Invitrogen and synthesized by Tsingke Biotechnology. The siRNA was transfected with NeoLNP RNA Transfection Kit (Scindy Pharmaceutical) according to the manufacturer’s instructions. After 6–8 h, medium was replaced with DMEM 2% FBS, and cells were cultured for an additional 24 h. The next day, cells were transfected again under the same conditions, followed by cell lysis. The efficiency of TRIM25 knockdown was determined by immunoblot with a specific TRIM25 antibody. The siRNA sequences used in this study are listed in Table S2.

### Real-time quantitative PCR analysis for gene expression

Total RNA was extracted from treated DEFs using TRIzol reagent (Invitrogen, USA). First-strand cDNA was obtained from extracted RNA, which was removed of genomic DNA according to the manufacturer’s instructions and reverse transcribed by the All-in-One First-Strand Synthesis MasterMix (BestEnzymes, China). Tissue samples were placed in TRIzol reagent and homogenized (M.P. Biomedicals, FastPrep-24). RNA extraction and the reverse transcription steps were the same as those of the cell samples. qPCR was performed using the F488 SYBR qPCR Mix (Universal) (BestEnzymes, China). The duck β-actin gene was used as an internal control gene to normalize the targeted gene expression value. The quantity of mRNA was calculated by the 2−ΔΔCt method and represented as the mean ± SEM (*n* = 3). DHAV-3 copy number determination in cells was performed according to the one-step TaqMan fluorescent quantitative RT-PCR method constructed in our laboratory. The relative transcript levels of target genes were analyzed using the 2−ΔΔCt method and compared with the blank control group. Primers employed are listed in Table S1.

### Ubiquitination assay

To evaluate the effect of duTRIM25 on IRF7 ubiquitination, HEK293T cells were co-transfected with IRF7-His, Ub-HA, and either TRIM25-Flag or an empty vector. At 24 h post-transfection, cells were treated with MG132 for 8 h before being harvested with IP buffer (Beyotime, China) containing protease inhibitor cocktail (MCE, China). Cell lysates were immunoprecipitated using anti-His mAb and analyzed by immunoblotting with anti-His, anti-HA, and anti-Flag antibodies. A similar protocol was employed to investigate the influence of TRIM25 on the ubiquitination of RIG-I.

### Luciferase reporter assays

DEFs cultured in 48-well plates were co-transfected with luciferase reporter plasmid and *Renilla* luciferase-expressing plasmid (pRL-TK, Promega, USA), along with the indicated expression plasmids, using PlusTrans (Nulen, CT801). Luciferase activity was measured using the Dual Luciferase Reporter Assay Kit (Vazyme, DL101-01) according to the manufacturer’s instructions. Results were expressed as relative firefly luciferase activities normalized to *Renilla* luciferase activities.

### Animal experiments

Age-stratified ducklings were utilized: 14-day-old for TMUV challenges (*n* = 10) and 3-day-old for NDRV and DHAV-3 infections (*n* = 10). PEI packaged plasmids (PEI: DNA ratio 1:2.5) were diluted in sterile 5% glucose and incubated for 30 min after mixing. At 24 h post-injection, ducks were intramuscularly inoculated with 10⁷ TCID_50_ TMUV/NDRV or 50 μL DHAV-3. Control groups received equivalent volumes of DMEM. At 3 days post-infection (dpi), three ducklings from each group were euthanized for sample collection from liver, spleen, lung, and brain tissues. For histopathological analysis, liver and spleen samples were fixed in 4% paraformaldehyde, embedded in paraffin, sectioned, and stained with hematoxylin and eosin (H&E) for examination by light microscopy. For the TCID_50_ assay, the livers, spleens, lungs, and brains from ducklings were lysed by a tissue grinder in PBS buffer. The viral RNA and IFN-β, Mx, and OASL expression were detected by RT-qPCR. For survival analysis, eight ducklings per group were monitored daily for 7 days following infection, and survival rates were recorded.

### Statistical analysis

All experiments were repeated at least three times independently. The data were analyzed with Student’s *t*-test and one-way analysis of variance (ANOVA) using GraphPad Prism (version 10.0.0). Data are presented as the means ± standard deviations (SD). For significant differences, a *P* value <0.05 was considered statistically significant.

## SUPPLEMENTAL MATERIAL

Supplemental material mbio.00470-26-s0001.docx Fig. S1 to S8; Tables S1 and S2.

ASM does not own the copyrights to Supplemental Material that may be linked to, or accessed through, an article. The authors have granted ASM a non-exclusive, world-wide license to publish the Supplemental Material files. Please contact the corresponding author directly for reuse.
