# Supplementary material for: TRIM25 promotes antiviral innate immune response by stabilizing IRF7 and its nuclear translocation
Source: mBio. 2026 Apr 1;17(5):e00470-26. doi: 10.1128/mbio.00470-26 (PMC13170311; doi:10.1128/mbio.00470-26)
Supplement: Supplemental material — Fig. S1 to S8; Tables S1 and S2. [file mbio.00470-26-s0001.docx]

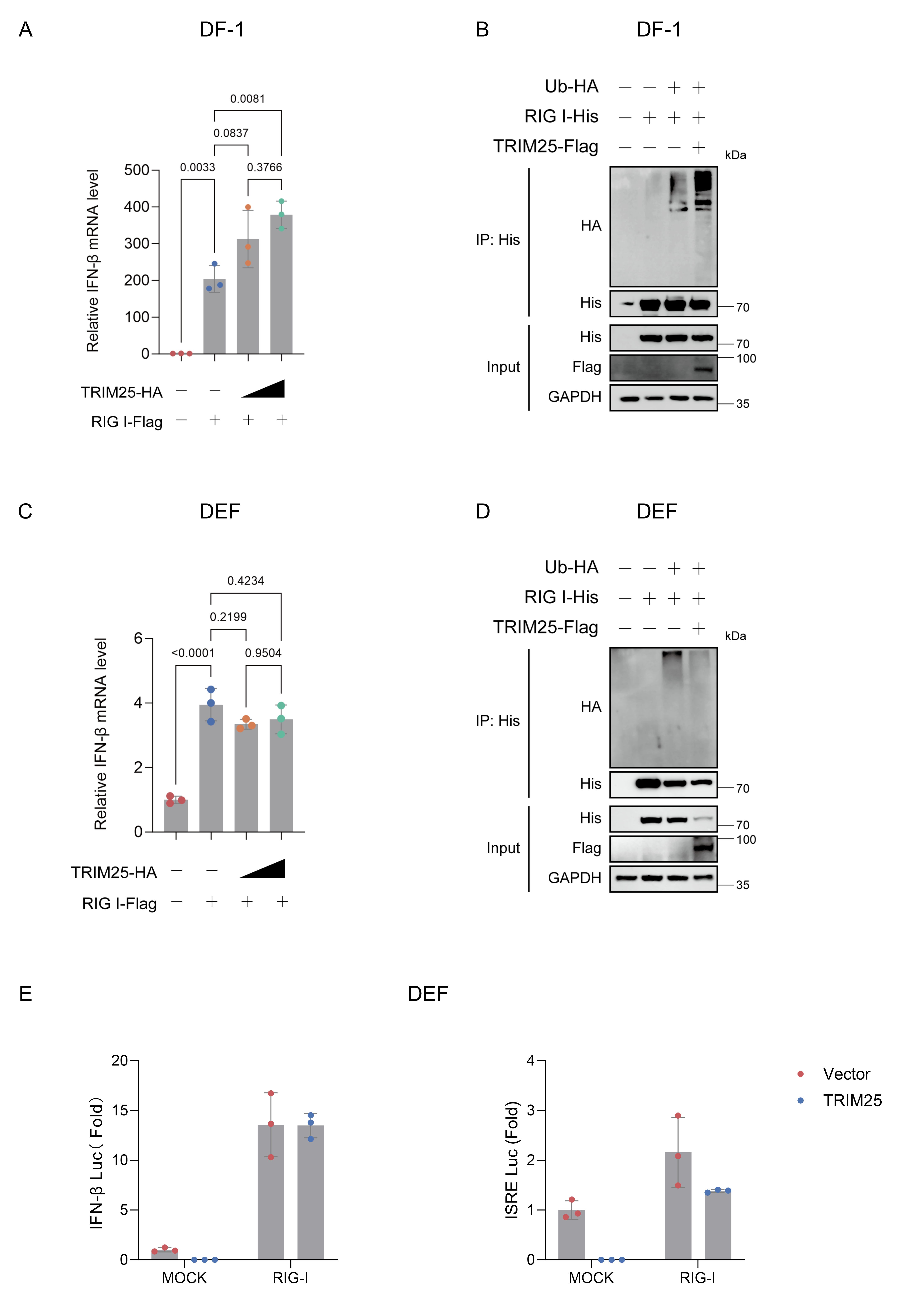


**Figure S1.** TRIM25 promotes duRIG-I-mediated IFNβ induction and ubiquitination in DF-1 cells

**A-D**. DF-1 (A, B) or DEFs (C, D) were co-transfected with RIG-I and increasing amounts of TRIM25 or empty vector. IFNβ mRNA levels (A, C) were determined by RT-qPCR at 36 h post-transfection (mean ± SD, n = 3). Statistical analysis was performed using one-way ANOVA with Dunnett’s multiple comparisons test. Co-IP assays (B, D) were performed using anti-His antibody to detect RIG-I ubiquitination.

**E.** Dual-luciferase assay of DEFs co-transfected with RIG-I, TRIM25, and reporter plasmids. Luciferase activity was normalized to Renilla luciferase.


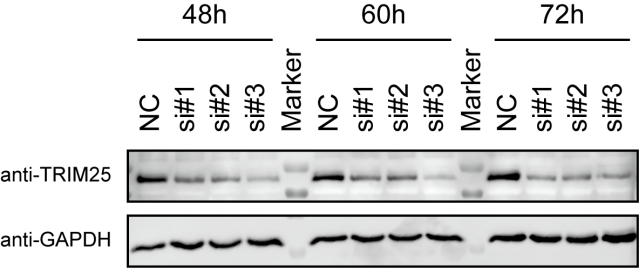


**Figure S2.** Gene silencing efficiency of siRNA targeting TRIM25 in DEFs.

DEFs were transfected with either negative control siRNA or siRNA targeting TRIM25. Western blot analysis to evaluate knockdown efficiency with an anti-duTRIM25 antibody.


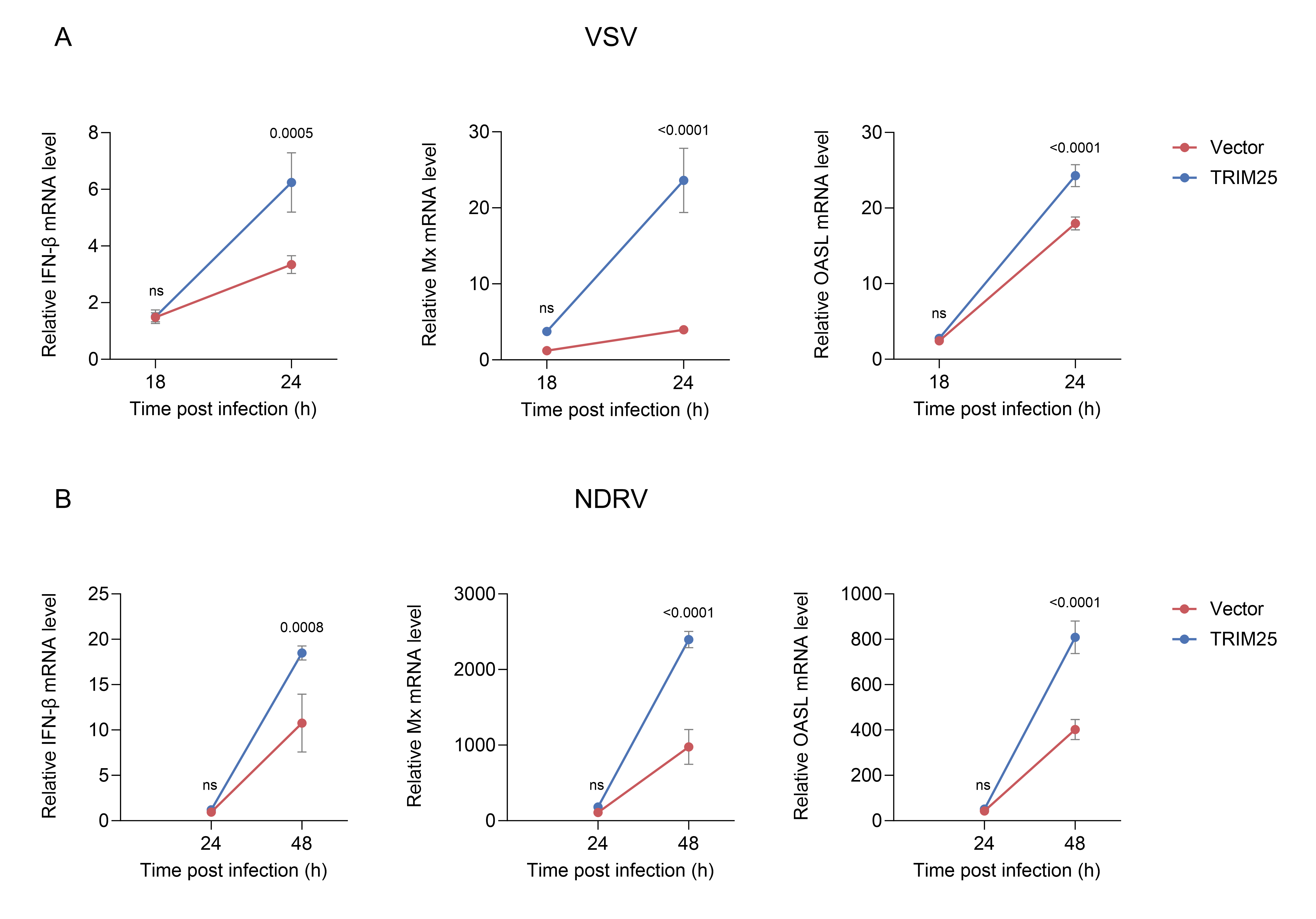


**Figure S3.** TRIM25 inhibits type I IFN production during RNA virus infection.

**A-B.** DEFs were transfected with TRIM25 and then infected with VSV (0.01MOI, A), NDRV (1000 TCID50, B). IFNβ, Mx, and OASL mRNA levels were quantified by RT-qPCR (mean ± SD, n = 3). Statistical analysis was performed using one-way ANOVA with Dunnett’s multiple comparisons test.


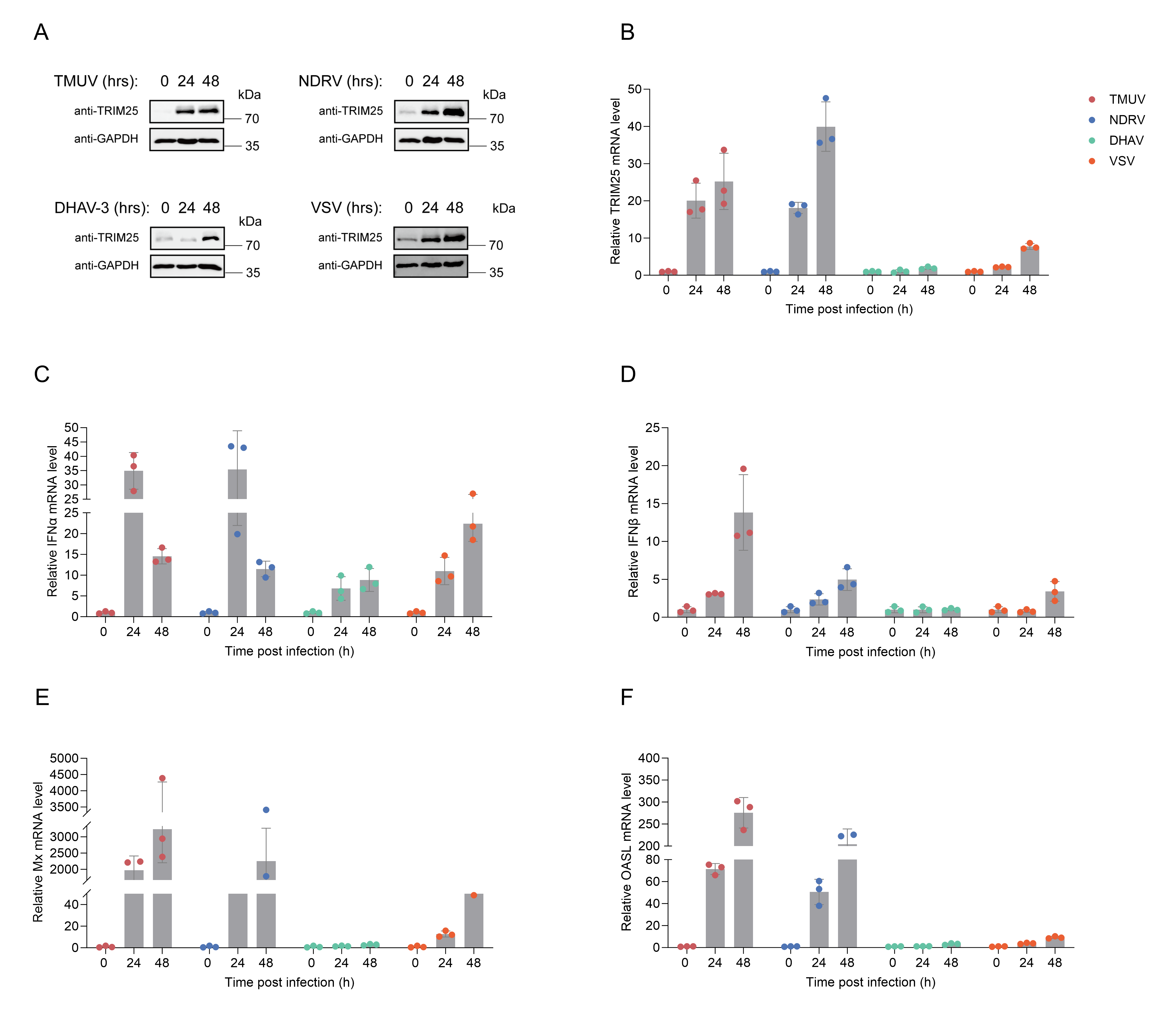


**Figure S4.** TRIM25 expression induced by RNA viral infection.

1. **F.** DEFs were infected with TMUV (2000 TCID50), VSV (0.01MOI), NDRV (1000 TCID50), and DHAV-3. The protein levels of TRIM25 (A) were detected via Western blot analysis. TRIM25 (B), IFNα (C), IFNβ (D), Mx (E), and OASL (F) mRNAs were measured by RT-qPCR at the indicated time points (mean ± SD, n = 3). Statistical analysis was performed using one-way ANOVA with Dunnett’s multiple comparisons test.


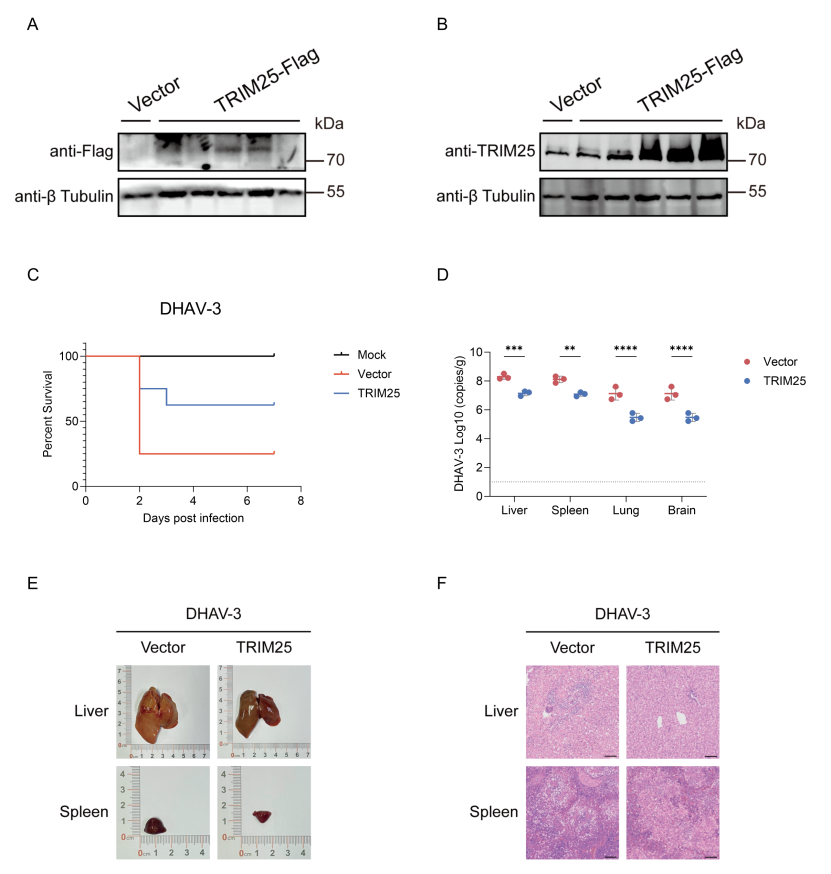


**Figure S5.** TRIM25 protected the duck from RNA viral infection in vivo.

**A-B**. Western blot analysis of TRIM25 expression in duck lungs 2 days post PEI-packaged TRIM25-Flag injection.

**C.** Survival curves (Kaplan–Meier) of ducks injected with TRIM25 or vector (n = 8 per group) and infected with DHAV-3. Survival was monitored for 7 days.

**D.** RT-qPCR detection of DHAV-3 RNA in liver, spleen, lung, and brain at 3 days post-infection.

**E.** Gross pathology of liver and spleen from TRIM25 and vector groups at 3 days post-infection with DHAV-3. The images are from one of three representative ducks.

**F.** Representative H&E-stained images of liver and spleen from TRIM25 and vector groups infected with DHAV-3 for 3 d. Scale bar, 100μm

Statistical analysis was performed using one-way ANOVA with Dunnett’s multiple comparisons test. **p < 0.01, ***p < 0.001, ****p < 0.0001


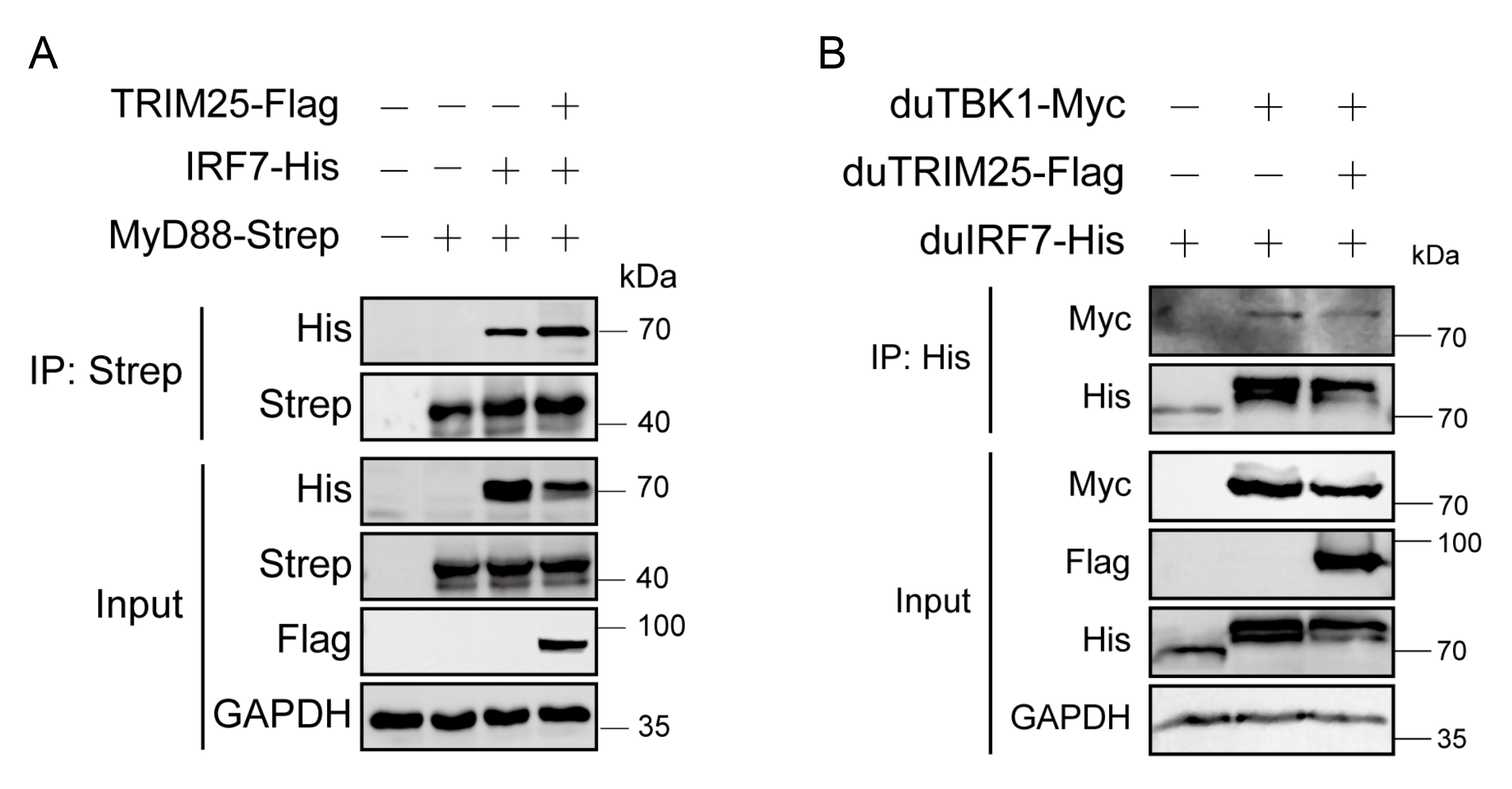


**Figure S6.** TRIM25 selectively facilitates IRF7-MyD88 interaction without enhancing IRF7-TBK1 complex formation.

A-B. CO-IP assays of HEK293T cells to detect the interaction of IRF7-MyD88 (A) and IRF7-TBK1 (B). Cells were co-transfected with MyD88-Strep or TBK1-Myc, IRF7-His, and TRIM25-Flag expression plasmids or an empty vector for 36 h and harvested for Co-IP using an anti-His primary antibody.

**
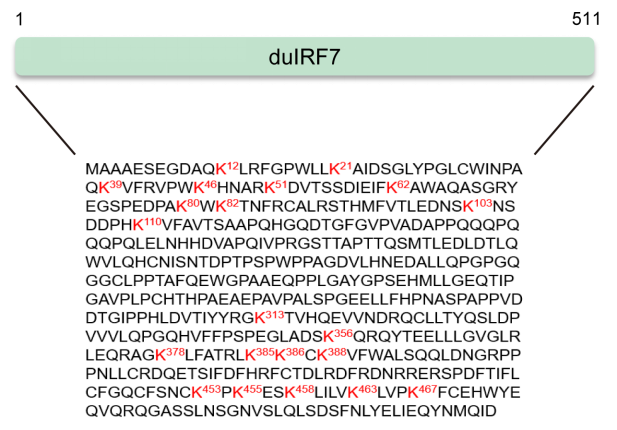
**

**Figure S7.** Schematic representation of Lysine residues on duIRF7.


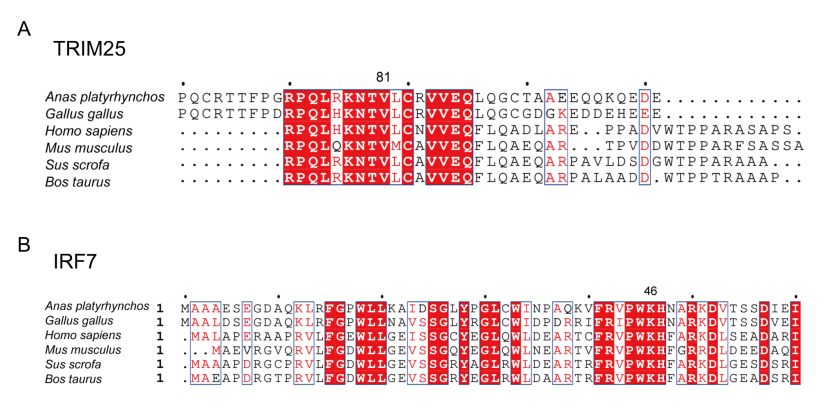


**Figure S8.** Protein sequences homology analysis.

**A-B.** TRIM25 (60-100aa, A) and IRF7 (1-60aa, B) from Anas platyrhynchos, Gallus gallus, Homo sapiens, Mus musculus, Sus scrofa, and Bos taurus were aligned using ESPript 3.0

Table S1. Primers used in this study.

| Primer Name | Primer Sequence (5’-3’) |
| --- | --- |
| duTRIM25-F | TGACCCGAGCCGTGT |
| duTRIM25-R | CTTTTTCATTTGGCAGAGGGAG |
| Pcdna3.1-duTRIM25-F | ACCGAGCTCGGATCCACTAGTCCAGTGTGGTGATGGCGGCGCTGACCCGAGC |
| duTRIM25-Flag-R | GTCGTCATCCTTGTAATCCTTTTTCATTTGGCAGAGGGAGAGAACAGT |
| pcdna3.1-dutrim25△RING-F | ACTAGTCCAGTGTGGTGGCCACCATGACCACCTTCCCGGGCCG |
| pcdna3.1-dutrim25△SPRY-R | GTCGTCATCCTTGTAATCATACTGCAAAAGCTCCT |
| q duTRIM25-F | CCACCCTCAGCGTTTCACCTATTGT |
| q duTRIM25-R | CTCCCGGTCCATGCTGCCATA |
| q TMUV E-F | AATGGCTGTGGCTTGTTTGG |
| q TMUV E-R | GGGCGTTATCACGAATCTA |
| q VSV G-F | TGCAAGGAAAGCATTGAACAA |
| q VSV G-R | GAGGAGTCACCTGGACAATCACT |
| q DHAV-3 VP3-F | GTGCTTAGACGCTGGCAGATT |
| q DHAV-3 VP3-R | TTCGATTGAAAACTATCTGAAACCTA |
| DHAV-3 VP3-Probe | FAM-TCAGTGGGCTAACACAGTGACCCCTG-BHQ |
| q duIFNα-F | TTGCTCCTTCCCGGACA |
| q duIFNα-R | GCTGAGGGTGTCGAAGAGGT |
| q duIFNβ-F | TCTACAGAGCCTTGCCTGCAT |
| q duIFNβ-R | TGTCGGTGTCCAAAAGGATGT |
| q duMx-F | TGCTGTCCTTCATGACTTCG |
| q duMx-R | GCTTTGCTGAGCCGATTAAC |
| q duOASL-F | TCTTCCTCAGCTGCTTCTCC |
| q duOASL-R | ACTTCGATGGACTCGCTGTT |
| q duβ-actin-F | GATCACAGCCCTGGCACC |
| q duβ-actin-R | CGGATTCATCATACTCCTGCTT |

Table S2. siRNA sequence of duTRIM25.

| Name | Sequence |
| --- | --- |
| siduTRIM25-1 | GGTGTATTGAGTGGTTTAA |
| siduTRIM25-2 | CAGCACTGAAGAAGAAACT |
| siduTRIM25-3 | GGAAGTAAGAAGAATGAAA |
